# Influence of Thermally Activated Crimped NiTi SMA Fibers on the Pure Shear Performance of Z-Shaped Mortar Specimens

**DOI:** 10.3390/ma19102059

**Published:** 2026-05-14

**Authors:** Eunsoo Choi, Jaloliddin Makhmudov, Jong-Su Jeon

**Affiliations:** 1 Department of Civil and Environmental Engineering, Hongik University, Seoul 04066, Republic of Korea; eunsoochoi@hongik.ac.kr; 2 Department of Civil and Environmental Engineering, Hanyang University, Seoul 04763, Republic of Korea; jongsujeon@hanyang.ac.kr

**Keywords:** crimped SMA fibers, shape memory effect, pure shear strength, shear ductility, thermal activation

## Abstract

**Highlights:**

- Crimped SMA fibers increased pure shear strength by 13–22%, depending on fiber content and heating.
- Thermal activation raised peak shear strength via recovery stress but reduced post-peak ductility.
- The 1.0% SMA fiber volume achieved the highest ductility index of 4.05 versus 1.03 for plain mortar.
- Crimped SMA fibers are viable reinforcement for shear-critical zones in seismic concrete structures.
- Thermal activation protocols must balance strength gain against potential cementitious matrix damage.
- A 1.0% fiber volume delivers the best balance of ductility and strength for energy-dissipating applications.

**Abstract:**

Concrete and cementitious composites exhibit brittle failure under shear stress, limiting their resilience in seismic and high-load applications; this study investigates whether crimped NiTi shape memory alloy (SMA) fibers can enhance pure shear strength and ductility of mortar specimens, with particular focus on the effect of thermal activation. Z-shaped mortar specimens were prepared with SMA fiber volume fractions of 0%, 1.0%, and 1.25%, tested under both non-heated and heated conditions using a Universal Testing Machine, with deformation monitored via LVDTs and Digital Image Correlation. SMA fiber reinforcement increased peak shear strength by 13% and 14.5% for 1.0% and 1.25% fiber volumes, respectively, under ambient conditions, reaching up to 22% enhancement after thermal activation due to recovery-stress-induced prestressing; the 1.0% fiber volume achieved the highest ductility index of 4.05 compared to 1.03 for plain mortar, while SMA fibers had negligible influence on initial shear modulus but substantially improved post-cracking response and crack bridging. These findings demonstrate that crimped SMA fibers effectively improve shear resilience of cementitious composites, with 1.0% fiber content offering the optimal balance between strength and ductility, though activation protocols require careful calibration to minimize thermal degradation of the matrix.

## 1. Introduction

Concrete and cementitious composite materials are widely used in structural engineering due to their high compressive strength and durability. However, they exhibit brittle behavior under shear stress, which makes them vulnerable to sudden failure in structures such as bridges, beams, and walls. Improving the shear strength and ductility of these materials is essential to provide structural resilience, especially in seismic zones and high-load conditions.

One promising solution to improve the shear strength and ductility of concrete and other cementitious composites is the incorporation of shape memory alloy (SMA) fibers [1]. SMA fibers possess the unique ability to recover their original shape when heated above a specific transition temperature, which can be used to provide adaptive reinforcement in concrete. When embedded in the concrete matrix, these fibers undergo phase transformations (martensite and austenite phases) that enable the material to actively resist shear forces by increasing stiffness under stress. This dynamic response improves the energy dissipation capacity of the structural element, delays crack initiation, and enhances overall structural stiffness. Additionally, SMA fibers contribute to the self-centering properties of concrete by recovering from micro-damage caused by shear stress [2,3].

### 1.1. Bond Behavior of SMA Fibers in Cementitious Matrices

The bond behavior between SMA fibers and cementitious matrices plays a critical role in the mechanical response of reinforced composites under shear-dominated loading conditions. Previous studies have extensively investigated this bond behavior, focusing on the potential of the SMA fibers to strengthen the structural performance of concrete members. Crimped SMA fibers, particularly cold-drawn types, demonstrated higher bond resistance than straight fibers due to mechanical anchoring from their geometric indentations. Among these, shorter-wavelength fibers consistently showed superior stiffness and peak bond strength [4]. Furthermore, SMA wires wrapped around concrete specimens improved bond strength through active confinement resulting from the shape memory effect, which effectively transformed failure modes from brittle splitting to ductile pull-out. This process improved energy dissipation under monotonic and cyclic loading [5,6].

Choi et al. investigated the bond behavior between concrete and reinforcing bars using cold-drawn, crimped NiTi SMA fibers finding that adding up to 1.5% SMA fibers improved bond strength by 47.9% and reduced radial strain by over 54% [7]. Pull-out tests further showed that heating the embedded SMA fibers induced recovery stress and expanded the fiber diameter, thereby exerting active confining pressure on the surrounding concrete matrix and improving both bond strength and composite durability [8,9]. Additionally, the bond-slip characteristics and frictional coefficients from experimental and finite element analyses showed the critical influence of fiber geometry, particularly wave-depth-to-wavelength ratio (DLR), on bond effectiveness and fiber-matrix interaction [4]. These findings collectively highlight the significant potential of crimped SMA fibers to enhance bond strength, stiffness, and ductility, motivating further investigation into their performance under pure shear conditions in specialized geometries such as the Z-shaped shear specimens used in the present study.

### 1.2. Thermal Activation and Recovery Stress Mechanisms

In addition to their bond-enhancing capabilities, SMA fibers also provide a unique mechanical advantage through their ability to generate recovery stress when thermally activated, thereby introducing an internal prestressing effect in cementitious elements. This active mechanism has been shown to significantly improve flexural performance, not only by resisting crack propagation but also by restoring structural integrity after damage. Experimental investigations on mortar beams reinforced with cold-drawn NiTi SMA fibers revealed that thermal activation led to substantial deflection recovery and load capacity improvements when special geometries designed to localize recovery stress were employed [10,11,12]. When activated by heating, these fibers showed outstanding performance, including prestressing effects, crack-closing capacity, and adequate bond strength to enable crack bridging, all of which contribute to improved concrete performance [13,14]. More importantly, recovery stress, rather than crack closure alone, was identified as the primary contributor to post-crack flexural strength restoration, acting analogously to externally applied prestress [15]. Additional studies involving crimped SMA fibers confirmed that heating could enhance load-bearing capacity by over 15%, demonstrating that temperature-induced activation is critical for mobilizing the full mechanical benefits of SMA reinforcement [16].

Prestressing effects have also been verified in SMA-reinforced mortar beams, where localized heating of Fe-based SMA wires produced recovery stresses exceeding 400 MPa, resulting in upward deflection and compressive stress fields without mechanical jacking [17,18,19]. Fe-based SMA demonstrated effective prestress development: recovery stresses increased during cooling, reaching up to 315 MPa, and produced measurable improvements in ductility, stiffness, and delayed crack initiation in mortar beams [20]. In another study, the activation of Fe-SMA stirrups increased shear strength by approximately 7.6% and effectively reduced shear crack formation [21]. Notably, NiTiNb fibers exhibited superior residual stress retention at ambient conditions—up to eight times higher than NiTi fibers—and, when combined with proper crack repair techniques, these prestress effects are sustained beyond the activation phase, improving long-term flexural performance [11].

### 1.3. SMA-Reinforced Mortars Under Various Loading Conditions

Recent experimental studies have further expanded understanding of the mechanical advantages of SMA fibers in cementitious composites, particularly under compressive, cyclic, and dynamic loading conditions. Mortars reinforced with NiTi SMA fibers have exhibited significant strain-rate sensitivity, with enhanced energy absorption and ductility during impact loading, though with slight reductions in compressive strength attributed to altered interfacial behavior [22]. Under cyclic loading conditions, SMA fibers reduced volumetric and plastic strains, and enhanced cyclic stiffness, especially when thermally activated [23]. Investigations into martensitic SMA beams emphasized the significance of tension-compression asymmetry and neutral axis shifts under bending loads and provided insights for predicting structural behavior under complex loading scenarios [24,25]. Additionally, fiber geometry and aspect ratio were identified as critical factors influencing compressive strength, toughness, and deformation behavior, with crimped fibers proving particularly effective in post-cracking response due to their superior mechanical anchoring [26]. Moreover, SMA fibers under monotonic and cyclic compressive loading significantly improved crack bridging, particularly when heat-induced recovery stress was introduced [27]. Lastly, the studies on cyclic compressive loading conditions confirmed the superior performance of both crimped and dog-bone-shaped fibers, with thermally activated fibers providing notable improvements in cyclic peak strengths, damage control, and deformation resistance [28].

### 1.4. Theoretical Framework of Pure Shear

Building on these insights, the current study investigates the pure shear strength, deformation capacity, and damage mitigation under pure shear loading conditions, with particular attention to the role of thermally induced prestressing. Z-shaped mortar specimens reinforced with crimped SMA fibers were used, as this geometry is specifically designed to isolate shear behavior. Pure shear strength, in a theoretical sense, refers to the ability of a material to resist deformation solely under shear stress, without any normal stress components, and is a fundamental concept in materials science and mechanics.

### 1.5. Research Gap and Objectives

Investigating the pure shear behavior of Z-shaped specimens reinforced with crimped SMA fibers is important for advancing structural engineering, particularly in enhancing the resilience and durability of concrete structures. While recent studies have examined the shear behavior of Z push-off specimens reinforced by various fibers, such as steel and polypropylene, under different conditions, including elevated temperatures [29,30], there remains a notable gap in the literature concerning the impact of SMA fibers on pure shear performance.

Existing research has also explored the mechanical properties of SMA-reinforced composites, focusing on aspects such as flexural and impact resistance [31,32,33]. However, these studies have not specifically addressed pure shear conditions in special configurations. Although superelastic (austenitic) NiTi wire offers well-documented self-centering and energy dissipation benefits under seismic loading [20], martensitic wire was adopted in the present study to isolate the contribution of thermally induced recovery stress—an active prestressing mechanism that cannot be replicated by superelastic wire operating passively under mechanical load. Addressing this research gap is essential for optimizing the use of SMA fibers in structural applications, potentially leading to innovative reinforcement strategies that enhance the shear strength and overall performance of concrete structures.

The primary objective of this research is to investigate the impact of SMA fiber reinforcement on the pure shear behavior of cementitious specimens, with a particular focus on the effect of thermal activation. Specifically, this study aims to analyze the difference in pure shear strength between plain and SMA-reinforced specimens while examining how heating influences the overall shear response. Furthermore, this research compares the effects of different fiber volume fractions on both the peak shear strength and the subsequent post-peak behavior to determine the effectiveness of the reinforcement under varying configurations.

## 2. Materials and Methods

### 2.1. Preparation and Manufacturing of SMA Fibers

The production of the crimped SMA fibers followed a multi-stage process, beginning with the refinement of the raw wire followed by mechanical deformation. Cold-drawn wire was selected for this study because cold drawing induces work hardening and residual martensite that enhance the fiber’s tensile strength and recovery stress upon thermal activation, while the resulting crimped geometry provides superior mechanical bond resistance compared to straight or annealed wire configurations, as demonstrated in prior pull-out and bond characterization studies on the same fiber type [4,16]. *Ni*_50.47_-*Ti*_49.53_ wires were supplied by SE Co., Ltd., Yangsan-si, Gyeongsangnam-do, Republic of Korea. Initially, *Ni*_50.47_-*Ti*_49.53_ (at. %) wires with a diameter of 1.2 mm were prepared by cold drawing. After an initial annealing at 700 °C, a second cold drawing stage was applied to reach a 0.85 mm diameter, followed by additional annealing at 500 °C for an hour. Subsequently, the wire was cold drawn to a final nominal diameter of 0.8 mm, resulting in a cold-drawn SMA wire with an actual diameter of 0.815 mm; the corresponding area reduction ratio was 8.06%.

**Figure 1:**
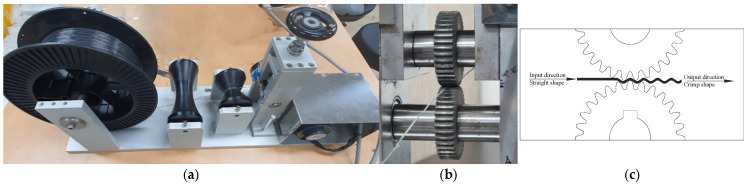
Manufacturing process of crimped SMA fibers: (**a**) device set-up for manufacturing crimped fibers; (**b**) rolling process; (**c**) rolling schematics.

The device set-up, as shown in Figure 1a, includes a continuous feed mechanism, a rolling device, and a traction system designed to maintain steady movement and controlled deformation. As part of this set-up, the previously described SMA wires are unspooled and guided into the rolling device where they pass through a set of precisely aligned interlocking rollers, as shown in Figure 1b. These rollers impart a sinusoidal corrugation onto the wires, as shown in Figure 1c. The deformation mechanism relies on meshing gear teeth that grip and reshape the wire under controlled pressure and a constant feed rate. As the SMA wire exits the rolling device, it is immediately pulled forward by a traction machine that ensures uniform tension, thereby preserving the imposed crimp geometry.

**Figure 2:**
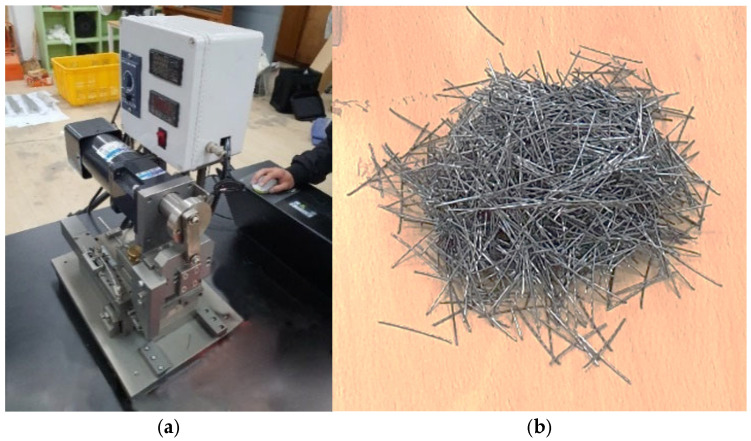
Cutting process and bundle of fibers. (**a**) Fiber-cutting machine. (**b**) Bundle of fibers.

Subsequently, the continuous crimped wire is cut into individual fiber segments using an automated cutting system. This system is equipped with a cutting ruler and synchronized feed mechanism to produce fiber segments with consistent lengths (see Figure 2a,b).

**Figure 3:**
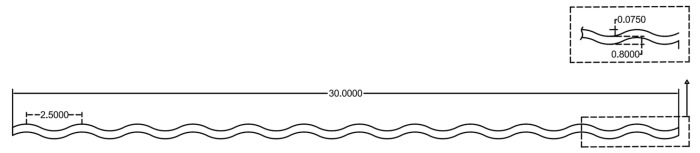
Dimensions of SMA crimped fibers (unit: mm).

Each fiber is manufactured to a target length of 30.0 mm, featuring a crimp pattern with a pitch of 0.8 mm, an amplitude of 0.075 mm, and an overall corrugated wavelength of 2.5 mm, as shown in Figure 3; the corresponding material properties for this single crimped SMA fiber, are presented in Table 1.

### 2.2. Phase Transformation and the Tensile Behavior of SMA Fibers

To verify the influence of the manufacturing process on the SMA behavior, the thermal properties were characterized. In general, cold-drawing work stabilizes the martensite phase resulting in a shift in the phase transformation temperatures [34,35]. Thus, this behavior was examined through Differential Scanning Calorimetry (DSC) curves of the as-received and cold-drawn SMA wires.

**Figure 4:**
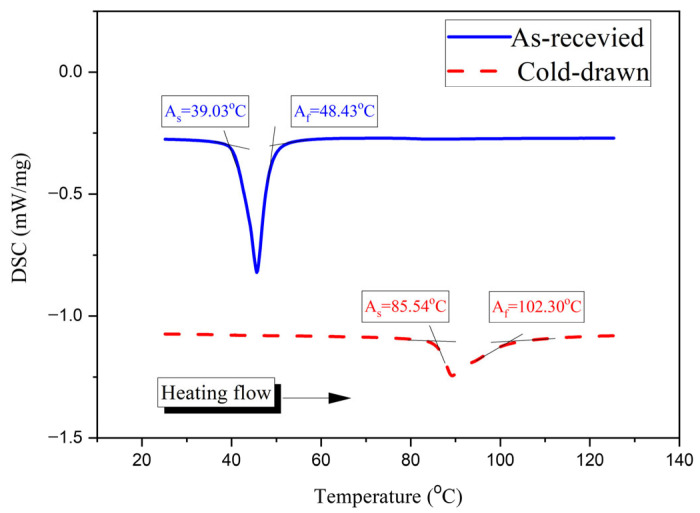
DSC curves of as-received and cold-drawn SMA wires.

Regarding the shape memory effect, the heat flow is significant for capturing the transformation temperatures from martensite to austenite [36]; consequently, Figure 4 shows two different DSC curves of the wires during the heating cycle. In the as-received wire, austenite transformation starts at 39.03 °C (*A_s_*) and finishes at 48.43 °C (*A_f_*). The cold drawing work shifted these temperatures to 85.54 °C (*A_s_*) and 102.30 °C (*A_f_*). This indicates that the cold-drawn SMA wire is more stable in the martensitic state at room temperature relative to the phase transformation.

**Figure 5:**
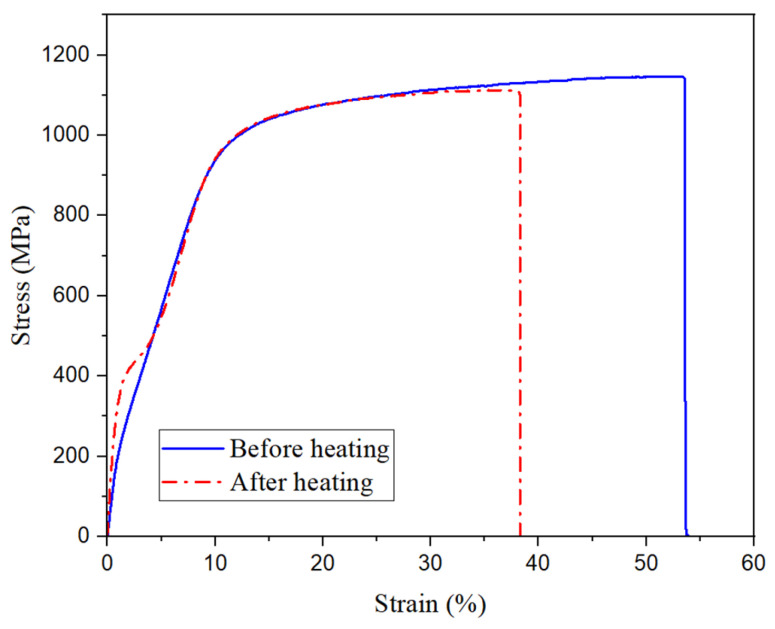
Stress–strain curve of the crimped SMA fibers before and after heating.

Figure 5 presents the tensile stress–strain curves of the crimped SMA fibers before and after heating. The 1% secant modulus (measured at 1% strain to capture the effective stiffness of the crimped geometry under initial loading) was 20.3 GPa for the non-heated fiber and 33.5 GPa for the heated fiber. This difference is attributable to geometric recovery: upon thermal activation above *A_f_*, the corrugated geometry partially straightens, reducing the compliance contribution of the crimp and producing a stiffer apparent response at low strain levels. Despite this geometric change, both conditions exhibited a consistent yield stress of approximately 980 MPa, indicating that the intrinsic martensitic strength of the wire is preserved through the heating process. The ultimate tensile stress was 1148 MPa for the non-heated fiber and 1113 MPa for the heated fiber—a modest reduction of approximately 3%—consistent with partial recovery of cold-work dislocations during heating, which marginally reduces the work-hardening plateau without compromising the fundamental load-bearing capacity of the fiber within the composite.

### 2.3. Mortar, Steel Reinforcement, and Specimen Preparation

To investigate the mechanical performance of the crimped SMA fibers under shear loading, reinforced mortar specimens were prepared using a Z-shaped test geometry. The specimens were designed with a central shear plane, enabling direct observation of bond performance and crack propagation under controlled loading.

**Figure 6:**
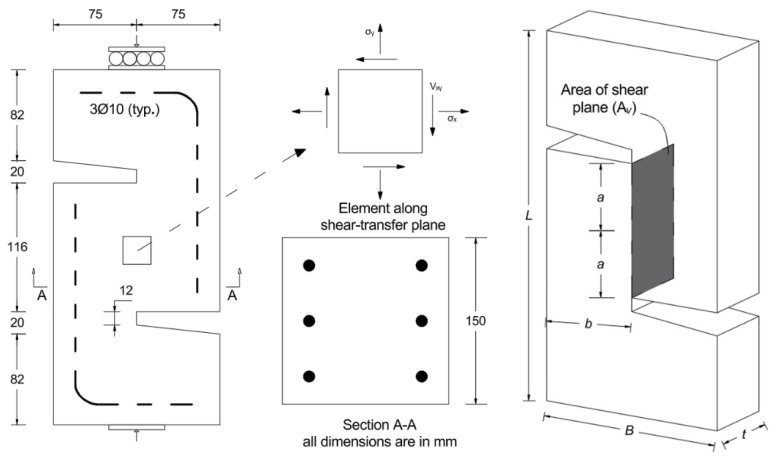
Details of the pure shear specimen; *a* = shear plane dimension, *b* = shear plane width, *A_v_* = shear plane area, *L* = specimen height, *B* = base length, *t* = base depth.

The specimen geometry consists of a rectangular body that incorporates a predefined shear notch, as shown in Figure 6. Reinforcement was provided in the form of L-shaped steel bars, as shown in Figure 6, to ensure that failure does not occur in those areas. The key shear zone, denoted as the shear plane area *A_v_*, was designed to concentrate stress and provide a controlled failure interface. The central shear plane is specifically configured to ensure that no normal stress components act on it, which ensures the stress state remains purely shear.

**Figure 7:**
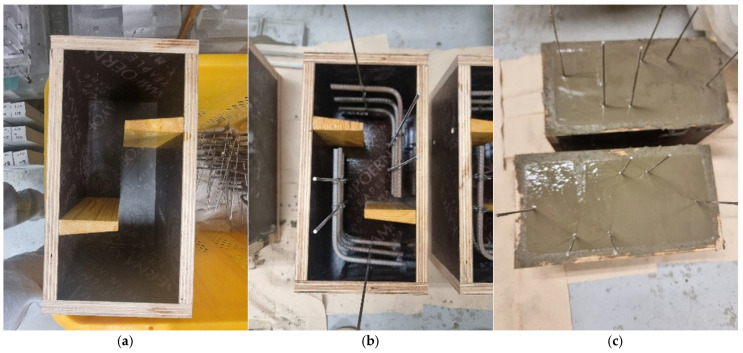
Manufacturing process of Z-shaped specimens. (**a**) Mold. (**b**) Arranged reinforcements. (**c**) Mortar casting.

Special wooden molds (see Figure 7a) were custom fabricated to match the specimen geometry, including spacers to form the voided region at the shear plane. Reinforcements were securely fixed in place before casting, ensuring consistency across all samples, as shown in Figure 7b. Crimped SMA fibers, where applicable, were uniformly distributed throughout the volume by manual dispersion during the mortar casting process.

The mortar matrix used for specimen casting followed a consistent mix design with a cement-to-binder ratio optimized for workability and durability. Portland cement was used as the primary binder, with fly ash incorporated as a supplementary cementitious material. Silica sand was added as a fine aggregate. The resulting mix achieved a compressive strength of *f*_c_′ = 40 MPa, as determined by standard cylinder tests (100 × 200 mm). The mix proportions are detailed in Table 2.

To quantify fiber distribution, two SMA volume fractions were used: 1.0% and 1.25%, while the reference specimen (0% SMA content) was prepared without fibers. The fiber volume fractions of 1.0% and 1.25% were selected based on evidence from prior experimental studies on the same crimped NiTi SMA fiber system, in which 1.0% is the most effective for shear and bond performance [7,16], and fractions exceeding 1.5% have been shown to produce adverse effects due to fiber clustering and reduced workability [4]. The 1.25% fraction was therefore chosen as a moderate intermediate step to examine whether an incremental increase in fiber content yields additional shear resistance while remaining within the practical dispersion limit. For each volume fraction, the total fiber volume, corresponding fiber length, and the number of individual fibers were calculated based on the geometry and weight of a single fiber, as shown in Table 3.

**Figure 8:**
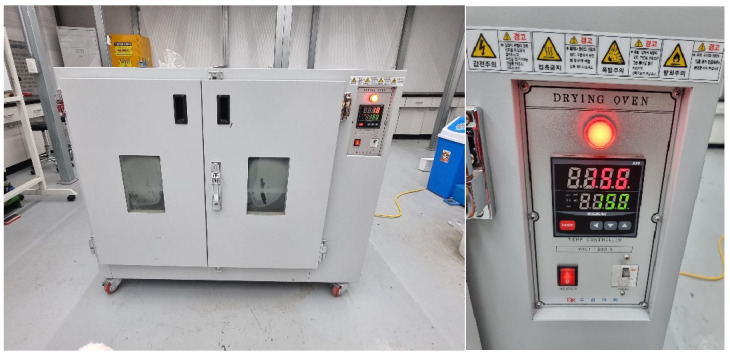
Activation of SMA.

The mortar was then poured into the prepared molds encapsulating both the steel reinforcements and the SMA fibers where applicable. The surface was leveled to ensure uniformity (see Figure 7c). All specimens were demolded after 24 h and then cured for 28 days to achieve consistent mechanical strength. After curing, the specimens were transferred to a drying oven (DreamScience, Suwon-si, Gyeonggi-do, Republic of Korea) (see Figure 8) to activate SMA fibers. The drying oven temperature was maintained at 150 °C, and specimens remained inside for 24 h. Based on prior experimental studies on the same crimped NiTi SMA fiber system, 150 °C was identified as the optimal activation temperature, ensuring complete phase transformation above *A_f_* while preserving matrix integrity [4,7].

### 2.4. Experimental Set-Up

**Figure 9:**
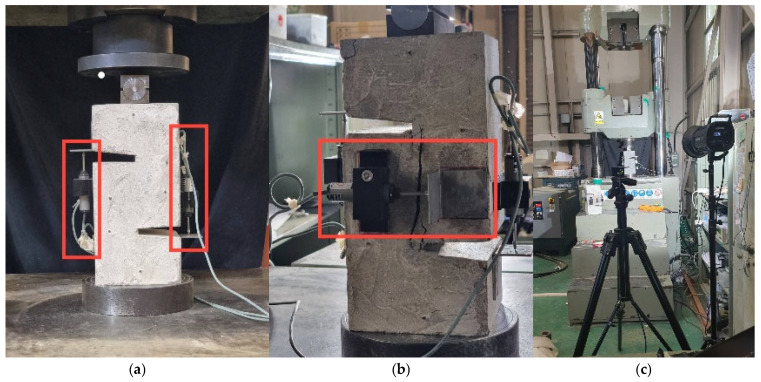
Installation of measuring devices. (**a**) Vertical LVDTs. (**b**) Lateral LVDTs. (**c**) DIC system.

To evaluate the pure shear behavior of mortar specimens with and without SMA fiber reinforcement, a carefully controlled test set-up was implemented using a Universal Testing Machine (UTM; SHINGANG PRECISION MFG. Co., Ltd., Seoul, Republic of Korea). To monitor deformation during loading, multiple Linear Voltage Displacement Transducers (LVDTs) were installed on the specimen. As shown in Figure 9a, vertically mounted LVDTs were placed on both sides of the specimen to measure vertical slip to capture the relative displacement between the upper and lower halves. This provides critical data on global shear deformation behavior under load.

In addition, as shown in Figure 9b, horizontally mounted LVDTs were positioned across the notched region to measure lateral (horizontal) slip—that is, the crack opening displacement as the shear crack propagates. This allows for precise monitoring of localized fracture behavior at the shear interface. Additionally, a Digital Image Correlation (DIC) was employed to capture minor crack propagation, which is not visible to the naked eye. As shown in Figure 9c, the experimental set-up included a camera system, lighting, and careful calibration to ensure accurate image tracking during loading. The DIC system was synchronized with the UTM and LVDTs to provide a detailed view of crack propagation and deformation patterns. All data, including load and displacement, were recorded with a sampling rate of 100 Hz, allowing for high-fidelity analysis of pure shear behavior.

### 2.5. Number of Specimens Tested

A total of 18 specimens were prepared, divided into 6 types and classified based on fiber content and thermal treatment conditions. The experimental matrix included reference specimens without SMA fibers, designated as Non-SMA-NH and Non-SMA-H, as well as specimens with 1.0% (SMA 1.0-NH and SMA 1.0-H) and 1.25% (SMA 1.25-NH and SMA 1.25-H) SMA fiber volume content. Specimens were tested under both heated and non-heated conditions to evaluate the thermally activated recovery behavior of SMA fibers. The full breakdown of specimen types and quantities is provided in Table 4.

## 3. Results and Discussions

### 3.1. Force–Displacement Curves and Shear Strengths

Each specimen type was evaluated using three samples, with the maximum applied shear forces determined from the force–displacement curves. Table 5 presents these individual values alongside their respective averages.

**Figure 10:**
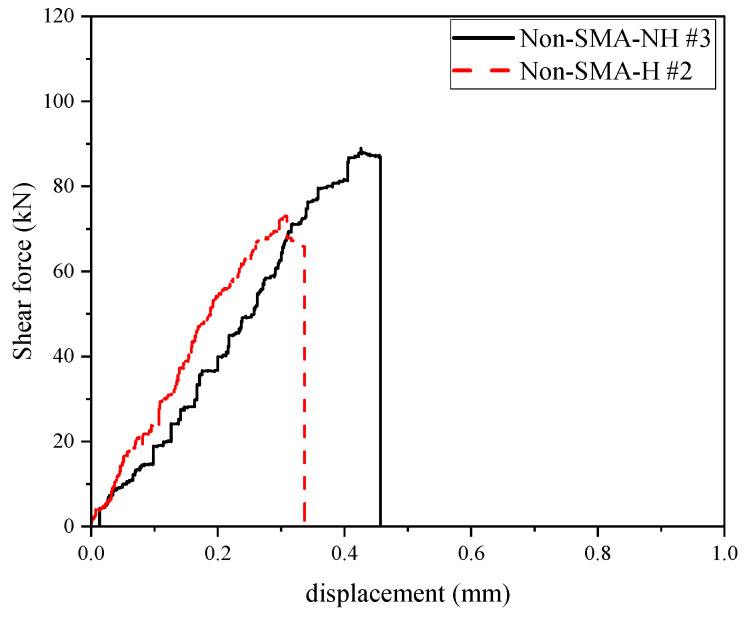
Shear force–displacement curve of representative specimens without SMA fibers.

**Figure 11:**
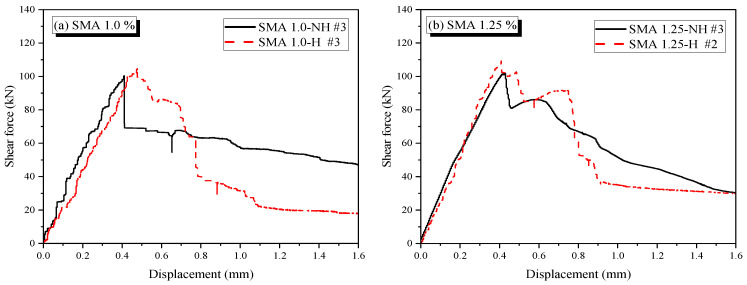
Shear force–displacement curves of representative SMA fibered specimens. (**a**) SMA fiber 1.0% specimens. (**b**) SMA 1.25% fiber specimens.

In this table, values in parentheses represent the shear stress, calculated by dividing the maximum force by the cross-sectional area of the specimen (116 mm × 150 mm = 17,400 mm^2^), as illustrated in Figure 6. Notably, the third sample of the Non-SMA-H group was excluded from the results as it failed to capture a valid response. Figure 10 and Figure 11 show representative force–displacement curves for each group, specifically selecting those specimens whose maximum values most closely align with the calculated averages.

The reference specimen, Non-SMA-NH #3 in Figure 10, exhibited a peak load of 88.9 kN at approximately 0.43 mm displacement, followed by an abrupt drop, indicating brittle failure. Thermal exposure of Non-SMA-H #2 specimen in Figure 10, further reduced both peak strength and displacement capacity, with the heated reference specimen reaching only 73.0 kN at 0.31 mm. The reduction in shear strength observed in the heated specimen may be attributed to the duration of thermal exposure. A previous study indicated that SMA fibers embedded in a 100 mm × 200 mm mortar cylinder reached 150 °C after 4 h of heating in an oven set to 150 °C [37]. Therefore, the 24 h heating duration applied in this study may have been excessively long, potentially causing microstructural damage within the matrix and resulting in the observed reduction in peak strength [38,39].

As shown in Figure 11a,b, the inclusion of SMA fibers prevented the abrupt failure observed in the pure mortar specimens, regardless of whether thermal activation was applied.

For the SMA 1.0 group, the inclusion of 1.0% crimped fibers improved both strength and ductility (see Figure 11a). The non-heated SMA 1.0-NH specimen reached 100.4 kN and exhibited a stable post-peak response. In contrast, its heated counterpart, SMA 1.0-H, achieved a higher peak strength of 104.6 kN due to the recovery stress of the SMA; however, it exhibited a relatively sharp post-peak decline. Specifically, while SMA 1.0-H maintained ductile behavior up to 0.7 mm beyond the peak load, it subsequently experienced a sudden drop in strength.

In the case of the 1.25% SMA fiber content, the heated specimen recorded the highest peak force of 108.2 kN, attributed to the thermally induced recovery stress. It maintained a stable displacement until approximately 0.75 mm, where an abrupt drop occurred (see Figure 11b). Similarly, the non-heated SMA 1.25-NH specimen reached 101.8 kN and maintained its load over a comparable displacement range, though it exhibited more stable degradation than the heated version.

The catastrophic failure observed in the heated SMA-reinforced specimens can be primarily attributed to the thermal degradation of the mortar matrix. While the embedded SMA fibers delayed total failure, they were insufficient to fully offset the loss of matrix load-carrying capacity. Another contributing factor is the alteration in the fiber geometry due to the shape memory effect. Upon heating, the corrugated parts of the fiber stretch, decreasing the corrugation height while the wire thickness increases due to Poisson’s effect [40]. While this maintains some bond resistance, the reduced geometric anchorage (corrugation height) likely diminishes the ability of the fiber to resist shear through dowel action, thereby compromising post-peak resistance. To quantify this adverse effect, the post-peak deformation range—defined as the difference between the maximum shear strain and the strain at peak stress— was computed for each group from the data in Table 6. For the 1.0% fiber group, this metric decreased from 1.08% (SMA 1.0-NH) to 0.51% (SMA 1.0-H), a reduction of 52.8%. For the 1.25% group, the post-peak deformation range contracted from 0.28% to 0.13%, a reduction of 53.6%. The near-identical proportional loss across both fiber volume contents indicates that the post-peak performance penalty is governed primarily by the thermal treatment of the matrix rather than by fiber content. Further evidence of matrix-level damage is provided by the plain mortar specimens: heating alone reduced the average peak shear strength of Non-SMA specimens by 17.4% (from 89.5 kN to 73.9 kN), while their ductility index remained at 1.03—confirming that thermal exposure degrades matrix load-carrying capacity without altering its inherently brittle failure character. For SMA-reinforced specimens, this matrix-level degradation compounds with the reduction in mechanical anchorage from decreased fiber corrugation height, producing the consistently sharper post-peak slope observed in all heated groups.

Regarding fiber volume content, the 1.25% specimens achieved higher shear strength gains compared to the 1.0% group, with increments of 3.4% and 1.2% for the heated and non-heated cases, respectively. However, their mechanical responses differed significantly. The 1.0% specimens exhibited a more progressive failure mode, with a post-peak deformation range (*γ*_max_ − *γ*_peak_) of 1.08% compared to 0.28% for the 1.25% specimens—a 3.9-fold difference—and a correspondingly steeper post-peak slope at the higher fiber content. This suggests that while increasing the fiber volume fraction from 1.0% to 1.25% yields a modest strength gain, it does not translate into improved post-peak performance. A possible explanation is that at higher fiber content, reduced fiber spacing increases the likelihood of adjacent fibers interacting within the same crack plane, which may compromise the ability of individual fibers to bridge cracks independently and sustain a stable pullout mechanism, leading to a more abrupt post-peak response.

### 3.2. Enhancement in Shear Strength

**Figure 12:**
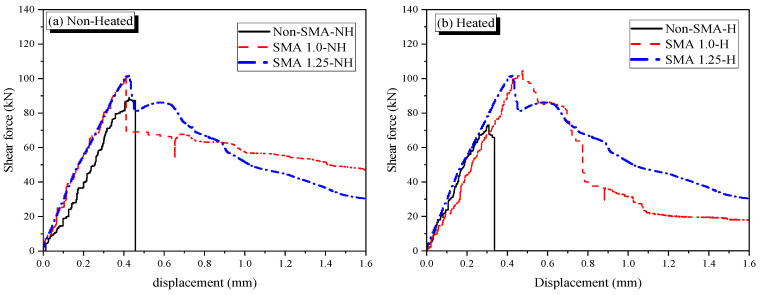
Comparison of shear behaviors of specimens according to activation. (**a**) Non-heated specimens. (**b**) Heated specimens.

Figure 12 compares the shear force–displacement curves according to activation of SMA fibers due to heating. Apparently, the heated cases shows higher increment in shear strength compared to the non-heated specimens. This study introduces an enhancement-ratio of shear strength to understand the effect of existence of SMA and the activation quantitatively.

**Figure 13:**
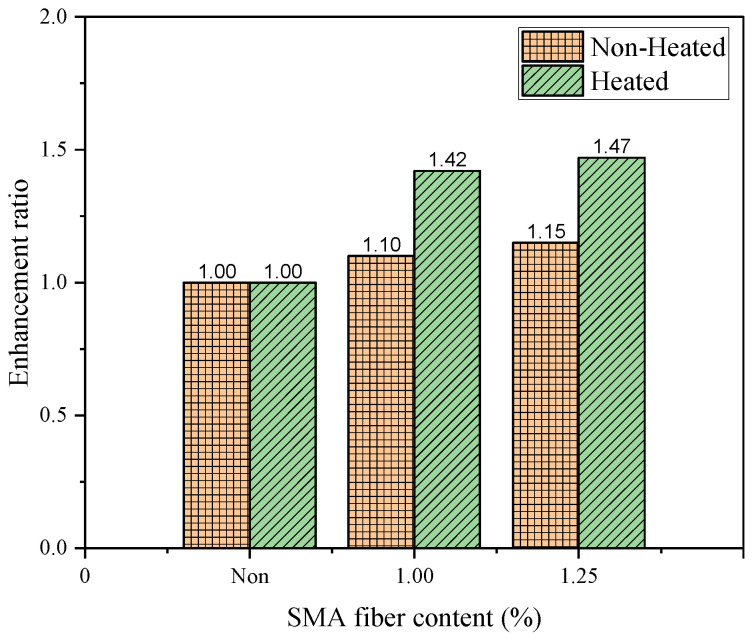
Enhancement ratio of shear strength.

Figure 13 presents the enhancement ratios of shear strength resulting from the embedment of SMA fibers. In this analysis, unreinforced specimens are used as the reference for both the heated and non-heated cases. Consequently, the enhancement ratio for the Non-SMA specimens is fixed at 1.0, and the ratios for the reinforced specimens are calculated based on the shear strengths listed in Table 5.

The enhancement ratios for the non-heated reinforced specimens are 1.10 and 1.15 for SMA 1.0-NH and SMA 1.25-NH, respectively. In contrast, the ratios for the heated reinforced specimens; namely, SMA 1.0-H and SMA 1.25-H, are 1.42 and 1.47, respectively. Although the heated specimens exhibited absolute shear strengths similar to those of the non-heated specimens (as shown in Figure 11), they demonstrated enhancement ratios approximately 28% higher than their non-heated counterparts. These results indicate that the thermal activation of SMA fibers is effective in increasing the shear strength of Z-shaped specimens, successfully compensating for the thermal degradation of the mortar matrix.

The reference specimens without SMA fibers provided the maximum shear stress of 5.11 MPa whereas SMA-1.0% and SMA-1.25% specimens (non-heated groups) showed 5.77 MPa and 5.84 MPa, respectively, corresponding to increases of 13% and 14.5%. The other groups of specimens reinforced with SMA fibers subjected to heating conditions demonstrated relatively higher shear stress capacities upon activation. The peak shear stress increased with SMA volume, reaching 6.22 MPa in the SMA-1.25% heated group while SMA-1.0% specimens showed a rise to 6.01 MPa after heating. The achieved higher strength—almost 22% increase, compared to non-heated reference specimens—can be attributed to the recovery stress generated by the phase transformation of the SMA fibers during heating. This prestressing effect increases resistance to crack opening under shear [13,16,17].

Based on an empirical model proposed in [30], the cracking shear strength $v_{cr}$ of mortar under pure shear, with a compressive strength of 40 MPa, can be estimated using Equation (1):

(1) $$v_{cr}=\beta \sqrt{f_{c}^{\prime }}$$

where *β* is the normalized cracking strength factor and $f_{c}^{\prime }$ is the compressive strength of concrete.

Taking *β* = 0.74 as proposed in [30], the predicted cracking shear strength is 4.68 MPa, which is close to the measured strength of the reference specimen. This value is lower than the peak strengths recorded for SMA-reinforced specimens, which reached up to 6.22 MPa. Compared to this theoretical baseline, the shear strengths of all tested specimens exceeded the predicted value. The SMA-reinforced specimens exhibited up to a 33% increase over the calculated cracking strength.

### 3.3. Shear Modulus

Based on the experimental data, the incorporation of crimped SMA fibers at 1.0% and 1.25% volume fractions did not cause any significant or consistent change in the shear modulus of the mortar specimens. While minor fluctuations were observed, the general trend indicates that the elastic shear stiffness of the matrix remains largely unaffected by the presence of SMA fibers.

In previous studies, SMA fiber reinforcement led to marginal changes in stiffness but substantial gains in ultimate strength and ductility under shear loading. Choi et al. [7] reported significant improvements in bond strength and crack resistance with crimped SMA fibers, without modifying the elastic behavior of the matrix. Similarly, Ji et al. [21] demonstrated that Fe-based SMA stirrups enhanced the peak shear capacity of reinforced concrete beams by approximately 7.6%, primarily due to recovery stress and improved crack control, while leaving the initial stiffness unaffected.

### 3.4. Ductility of Shear Strain

The influence of SMA fiber reinforcement on shear strain behavior was most evident in the post-peak response. While the strain at peak stress remained consistent across all specimen groups, only modest increases were noted in the SMA-reinforced specimens. However, the maximum shear strain values and the corresponding ductility indices indicate a substantial enhancement in post-peak deformation capacity due to the inclusion of SMA fibers. The ductility index (DI) was calculated as the ratio of the maximum shear strain to the shear strain at peak stress, expressed as in Equation (2):

(2) $$DI=\frac{\gamma_{\max}}{\gamma_{\mathrm{peak}}}$$

where *γ*_max_ is maximum measured shear strain before failure, and *γ*_peak_ is shear strain corresponding to peak shear stress.

**Figure 14:**
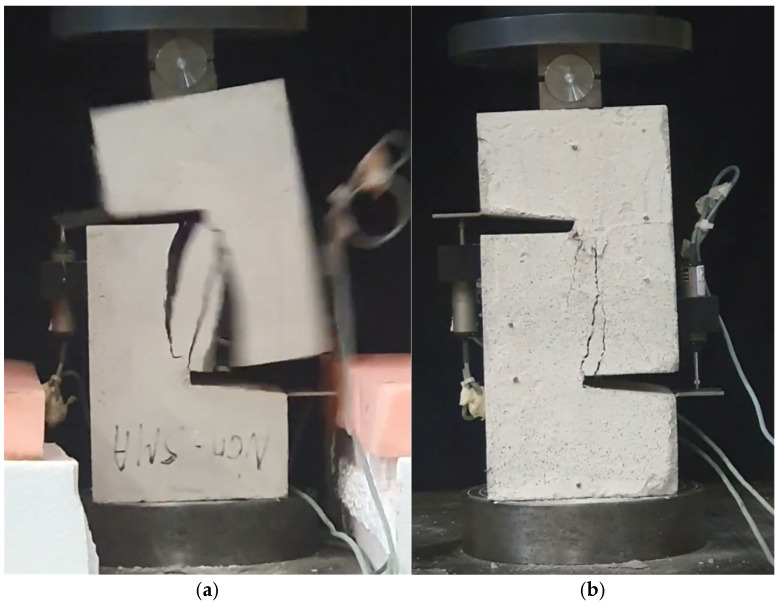
Visual comparison of failure modes. (**a**) Non-SMA-NH specimen. (**b**) SMA 1.0-NH specimen.

As summarized in Table 6, the control specimens exhibited limited deformation capacity, with a ductility index of 1.03, a brittle failure mode and minimal strain beyond peak stress (see Figure 14a). In contrast, all SMA-reinforced specimens showed markedly improved ductility. The non-heated SMA-1.0% group achieved the highest DI at 4.05, nearly four times that of the reference specimen, followed by the non-heated SMA-1.25% group at 1.66. Thermally activated specimens also outperformed the reference specimens in terms of ductility, though to a lesser extent than their non-heated counterparts. The heated SMA-1.0% and SMA-1.25% specimens reached ductility indices of 2.24 and 1.33, corresponding to 2.2- and 1.3-fold increases, respectively, over the reference mix. However, thermal activation reduced the ductility index by 44.7% for the 1.0% fiber group (from 4.05 to 2.24) and by 19.9% for the 1.25% group (from 1.66 to 1.33). The smaller percentage reduction at 1.25% should not be interpreted as greater thermal resilience: the non-heated baseline of that group was already 59% lower than for the 1.0% group, a consequence of fiber interference at higher volume content that promotes more abrupt post-peak failure even without heating, as discussed in Section 3.1. As already quantified in Section 3.1, the post-peak deformation range decreased by approximately 53% for both fiber groups after thermal activation, confirming that thermal activation impairs post-peak behavior to an equivalent degree at both volume contents. Overall, thermal activation consistently raises peak shear strength while approximately halving post-peak deformation capacity—a strength–ductility trade-off that must be carefully weighed in seismic applications; from this perspective, a 1.0% fiber volume offers the most practical balance for structures where energy dissipation governs design.

### 3.5. Failure Mode and Crack Propagation

Figure 14, and Table 7 and Table 8 illustrate the failure modes and crack propagation behavior of the specimens tested. The reference specimen without SMA fibers, as shown in Figure 14a, failed in a brittle and abrupt manner, characterized by sudden, complete separation along the shear plane. In contrast, SMA-reinforced specimens (see Figure 14b) demonstrated more favorable failure characteristics, such as gradual crack propagation, crack bridging, and partial structural continuity beyond peak load.

The DIC results shown in Table 7 and Table 8 capture the full evolution of strain and cracking across four loading phases for each specimen type. All SMA-reinforced specimens exhibited gradual crack development regardless of fiber content or thermal activation. The DIC system successfully captured the evolution of strain in these specimens across all loading phases. For the reference specimens (Table 7), the failure occurred abruptly; therefore, the DIC camera was unable to detect early-stage microcracks prior to fracture, resulting in limited visible crack development before final separation.

Unlike earlier studies [41,42], which involved beam specimens tested under three-point bending conditions and reported mode shifts from shear to flexural behavior due to thermal activation, no such transition was observed in this study. This is attributed to the geometry of the specimen, which was specifically designed to induce pure shear failure. As a result, thermal activation did not lead to a change in failure mode in any of the SMA-reinforced specimens (see Table 8).

Although thermal activation of SMA fibers increases peak shear strength, it consistently results in reduced post-peak ductility, consistent with the crack propagation patterns captured by DIC. This aligns with the interacting degradation mechanisms detailed in Section 3.1, and suggests the need for further exploration of their combined impact under pure shear conditions. A dedicated numerical investigation focusing on stress distribution within the fiber-matrix interface, fiber orientation effects, and the loading state of SMA fibers during thermal activation is being pursued by the authors to further elucidate these mechanisms.

## 4. Conclusions

This study experimentally investigated the pure shear behavior of Z-shaped mortar specimens reinforced with crimped SMA fibers, with particular emphasis on the influence of fiber volume fraction and thermal activation. Based on the experimental results, the following conclusions can be drawn:

- The addition of crimped SMA fibers increased the peak shear strength by 13% and 14.5% for fiber volumes of 1.0% and 1.25%, respectively, under ambient conditions. This enhancement reached up to 22% following thermal activation.
- Peak shear capacity scaled with fiber content, with 1.25% SMA-reinforced specimens achieving the highest shear stress of 6.22 MPa after thermal activation.
- Activation of SMA fibers triggered recovery stresses that acted as internal prestressing, raising peak strength but inducing more abrupt post-peak failure due to matrix degradation and fiber geometry alterations.
- SMA fibers had negligible influence on the initial shear modulus, confirming that their primary structural contribution occurs post-cracking via crack bridging and stress redistribution.
- The specimens with SMA fibers of 1.0% volumetric content achieved the highest ductility index of 4.05. While plain mortar failed brittlely, SMA-reinforced specimens transitioned to a progressive failure mode with maintained structural continuity.

The results demonstrate that crimped SMA fibers are effective in improving the shear strength, ductility, and crack control of cementitious materials under pure shear loading. From a practical standpoint, the 1.0% SMA fiber volume represents the most viable option for shear-critical zones in seismic concrete structures such as beam-column joints and shear walls. Regarding durability, Thomas et al. [22] investigated NiTi SMA fiber-reinforced mortars exposed to aggressive chemical environments and reported that the fibers remained free from corrosion, exhibiting superior durability in cementitious matrices. However, NiTi SMA fibers carry a significantly higher material cost than conventional steel or polypropylene fibers, and their wider adoption remains constrained by this economic barrier alongside the limited transfer of laboratory findings to construction practice [43]. To enhance structural performance, future investigations should prioritize the multi-scale optimization of the fiber-matrix interface, particularly identifying the optimal crimp geometry defined by the amplitude-to-wavelength ratio to maximize mechanical interlock while preventing localized matrix crushing during phase transformation. The competitive relationship between SMA-induced recovery stress and thermal degradation of the cementitious microstructure requires precise quantification to establish effective activation protocols. Furthermore, the long-term stability of the shape memory effect and recovery stress retention under cyclic thermomechanical loading must be addressed before large-scale structural deployment can be confidently recommended.

## Figures and Tables

**Table 1:** Specifications of an SMA fiber (per one unit).

Property	Value	Property	Value
Designation	CR25-075	Cross-sectional area (mm^2^)	0.50265
Diameter (mm)	0.8	Wavelength (mm)	2.5
Length (mm)	30	Wave height (mm)	0.075

**Table 2:** Mortar mix ratio.

Portland Cement	Fly Ash	Silica Sand	Water
1.00	2.00	0.45	0.15

**Table 3:** SMA fiber content in specimens.

Fiber Volume (%)	Total Volume (mm^3^)	Total Length (mm)	Quantity (EA)
1.00	68,400	136,077.48	4536
1.25	85,500	170,096.85	5670

**Table 4:** Experimental specimen matrix.

№	Specimen Type	Heating Condition	Fiber Volume (%)	Quantity (EA)
1	Non-SMA-NH	Non-Heating	0	3
2	Non-SMA-H	Heating	0	3
3	SMA 1.0-NH	Non-Heating	1.0	3
4	SMA 1.0-H	Heating	1.0	3
5	SMA 1.25-NH	Non-Heating	1.25	3
6	SMA 1.25-H	Heating	1.25	3

**Table 5:** Maximum applied force and shear stress.

Specimen	Non-SMA-NH	Non-SMA-H	SMA 1.0-NH	SMA 1.0-H	SMA 1.25-NH	SMA 1.25-H
	(kN/MPa)					
#1	87.3/(5.02)	74.8/(4.30)	92.2/(5.30)	103.4/(5.94)	100.9/(5.80)	106.1/(6.10)
#2	92.2/(5.30)	73.0/(4.20)	103.5/(5.95)	107.9/(6.20)	104.7/(6.02)	108.2/(6.22)
#3	88.9/(5.11)	N/A	100.4/(5.77)	104.6/(6.01)	101.8/(5.85)	111.4/(6.40)
Average	89.5/(5.14)	73.9/(4.25)	98.7/(5.67)	105.3/(6.05)	102.5/(5.89)	108.6/(6.24)

**Table 6:** Ductility indices.

Specimens	Strain at Peak Shear Stress (%)	Maximum Shear Strain (%)	Ductility Index
Non-SMA-H	0.26	0.27	1.03
Non-SMA-NH	0.35	0.36	1.03
SMA-1.0%-H	0.41	0.92	2.24
SMA-1.0%-NH	0.35	1.43	4.05
SMA-1.25%-H	0.39	0.52	1.33
SMA-1.25%-NH	0.42	0.70	1.66

**Table 7:** Failure modes and crack propagation for reference specimens.

Specimen	Phase 1	Phase 2	Phase 3	Phase 4
Non-SMA-NH	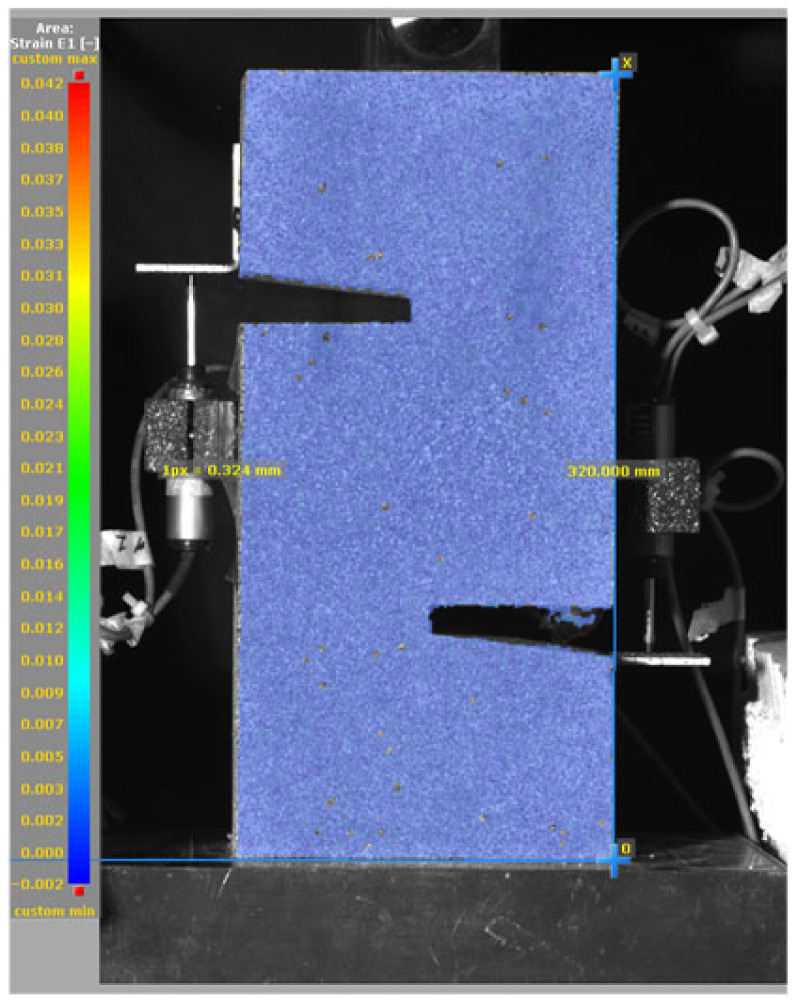	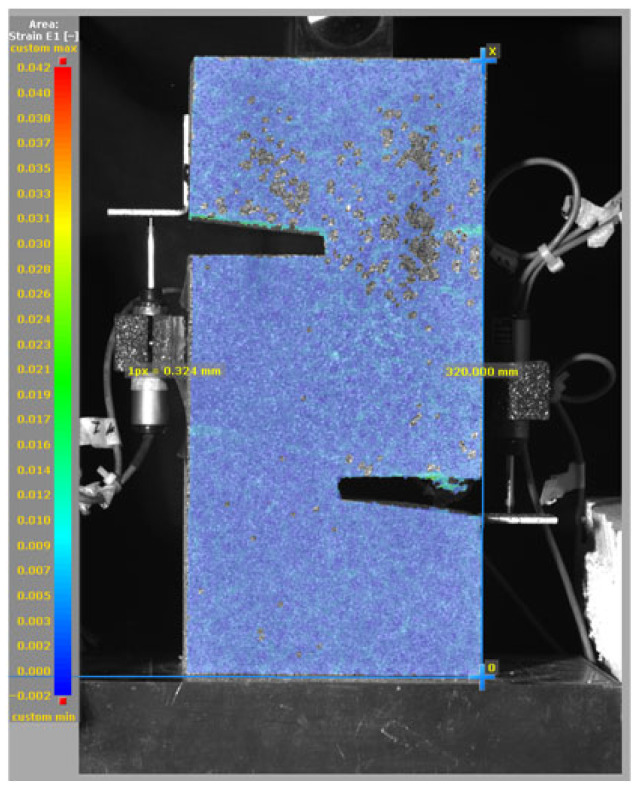	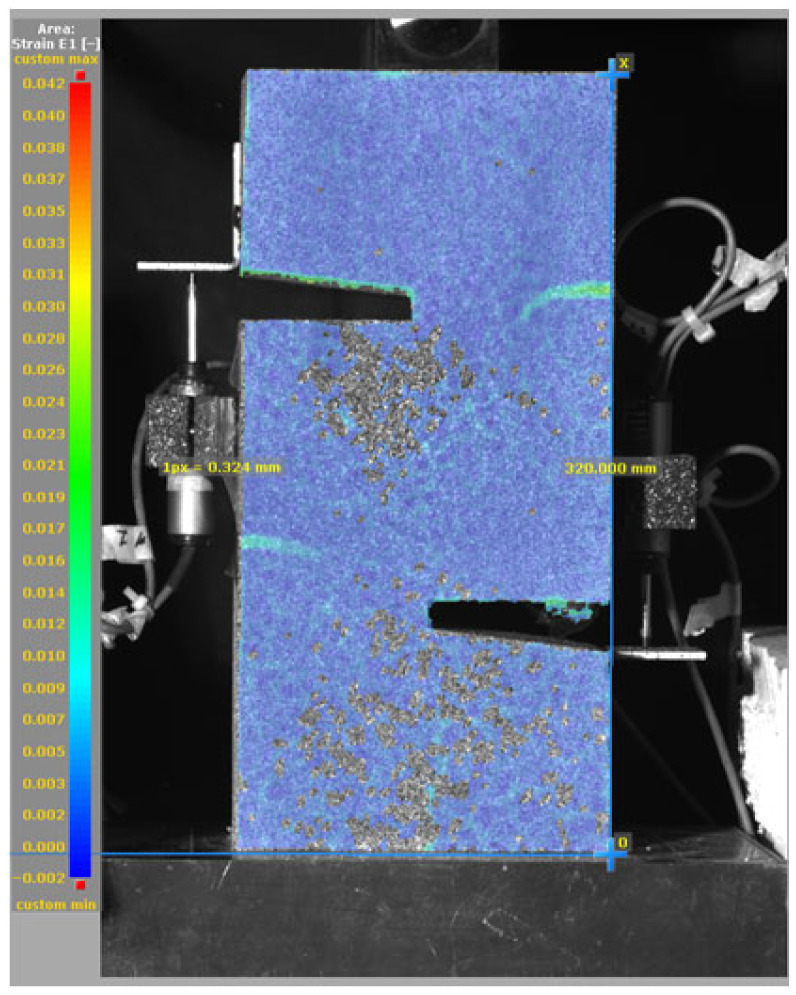	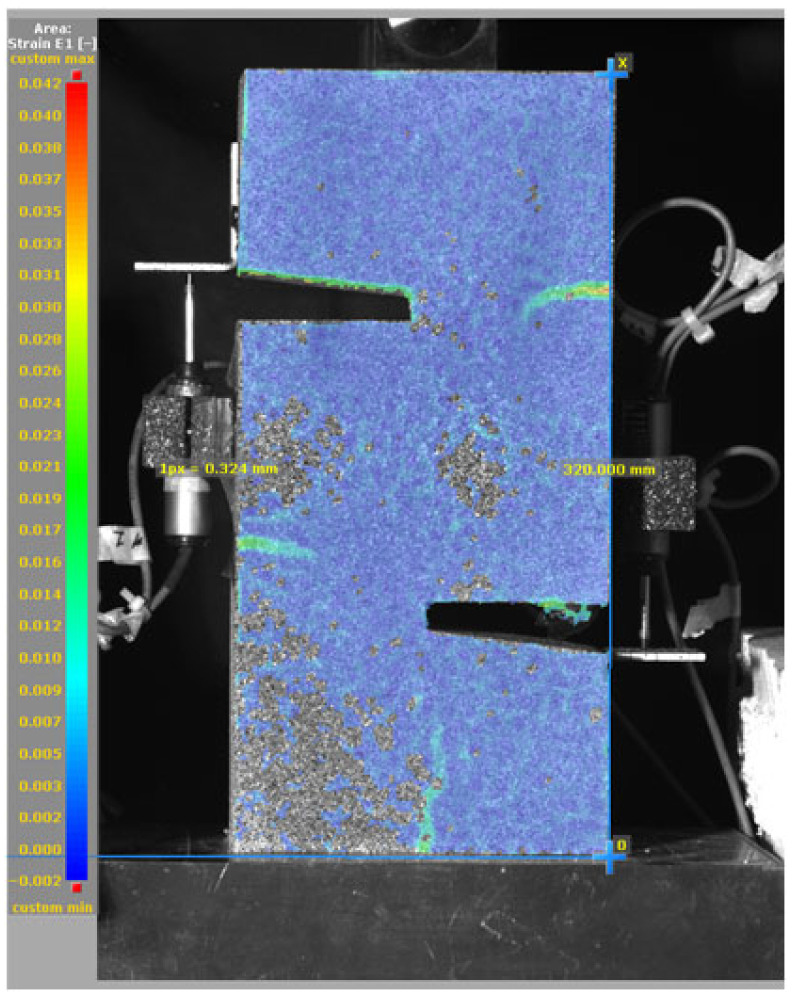
Non-SMA-H	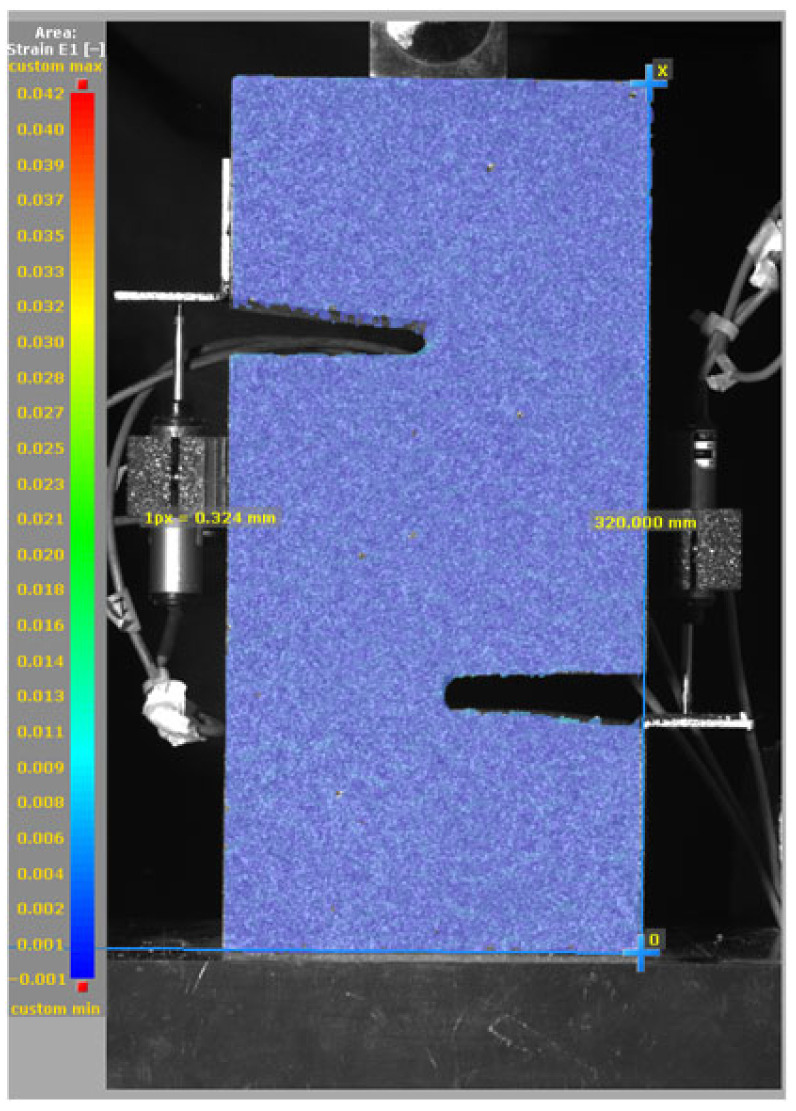	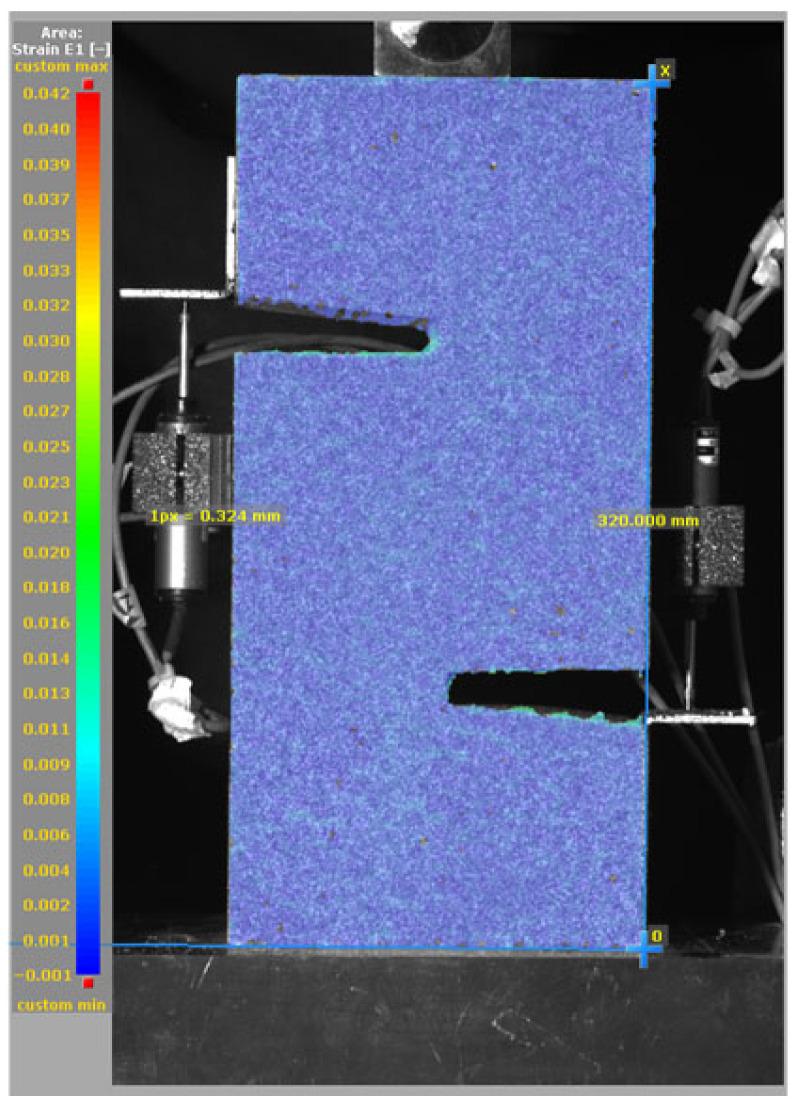	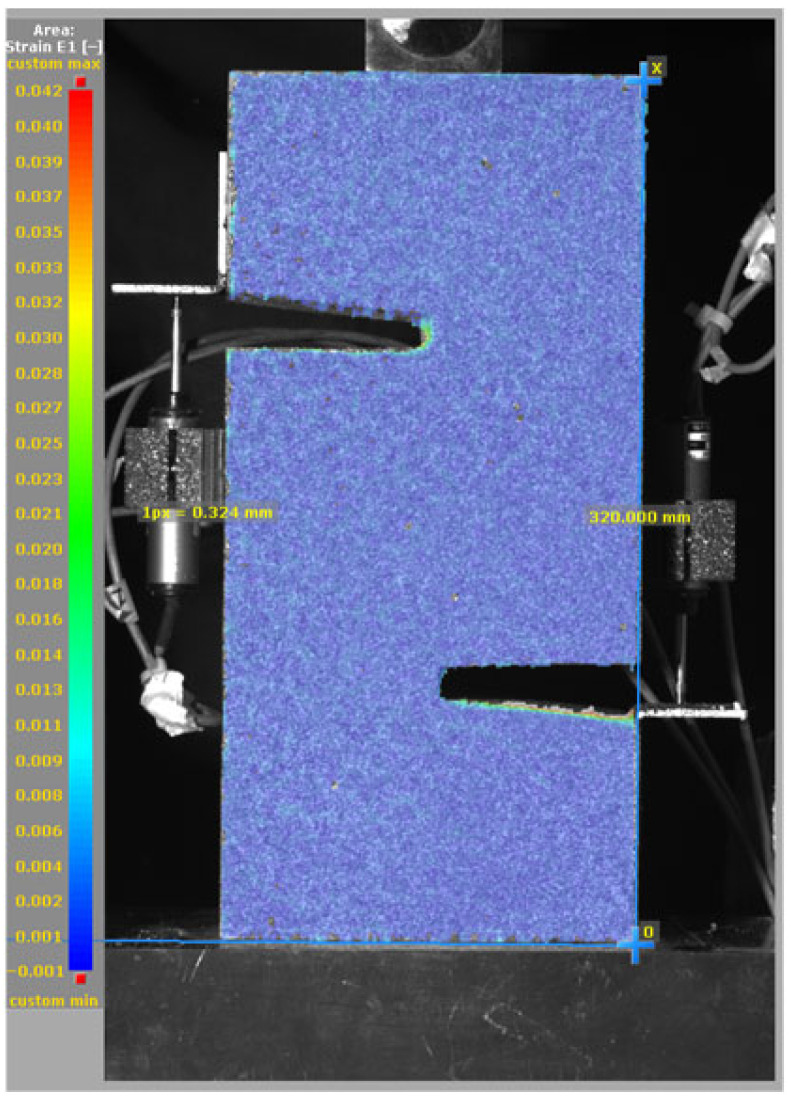	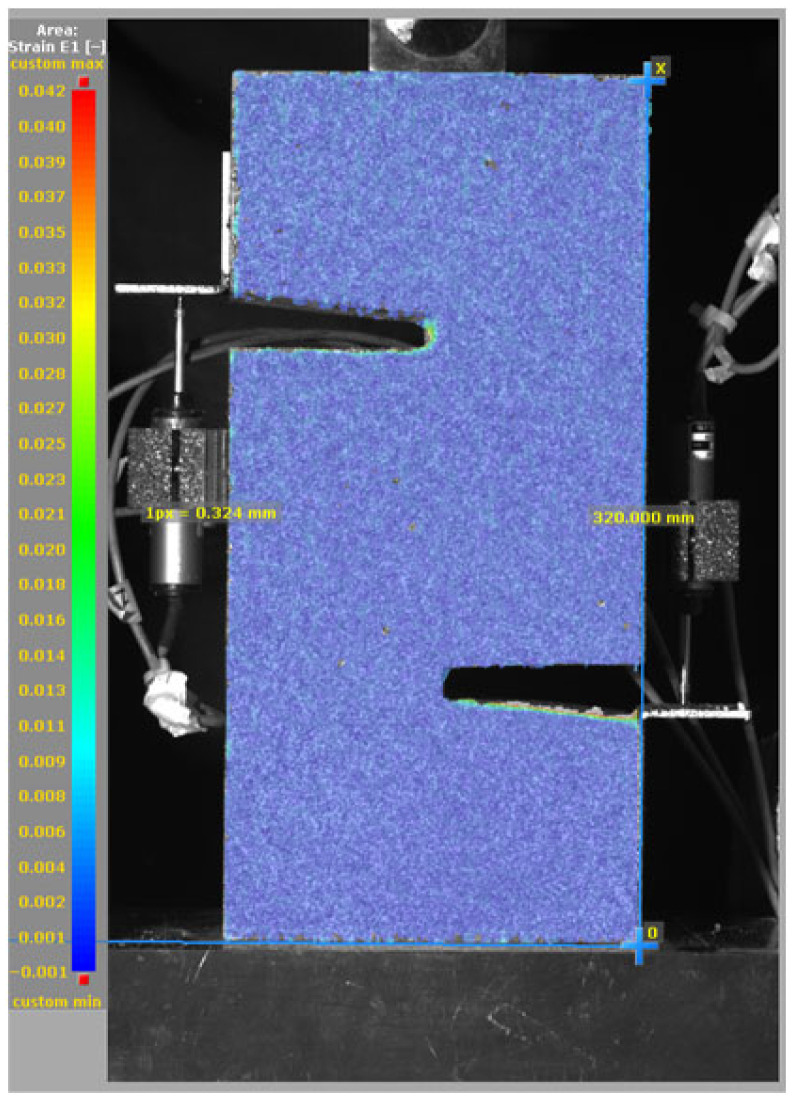

**Table 8:** Failure modes and crack propagation for SMA-reinforced specimens.

Specimen	Phase 1	Phase 2	Phase 3	Phase 4
SMA-1.0%-NH	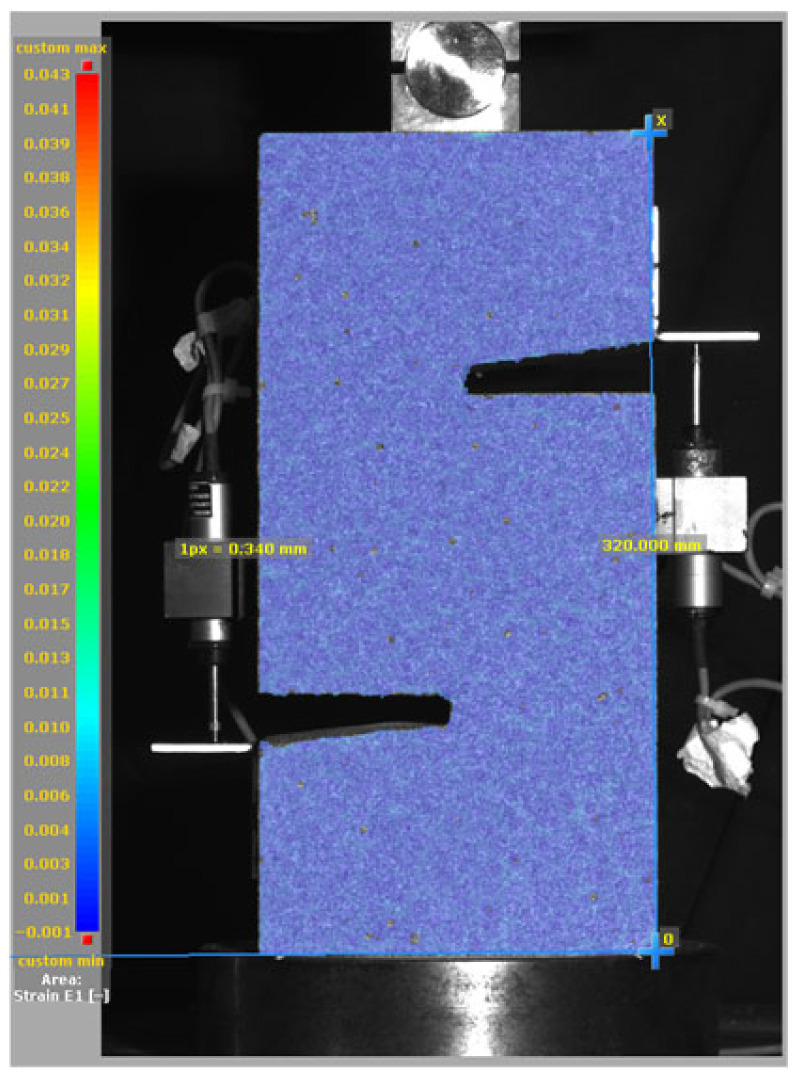	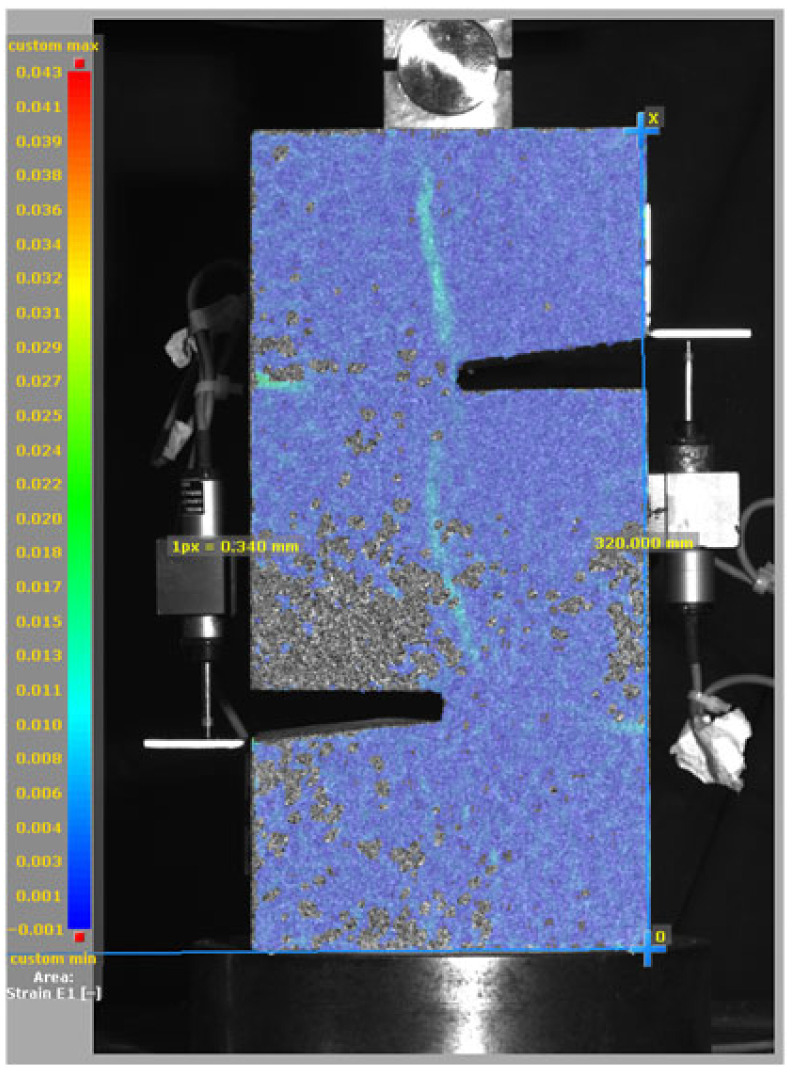	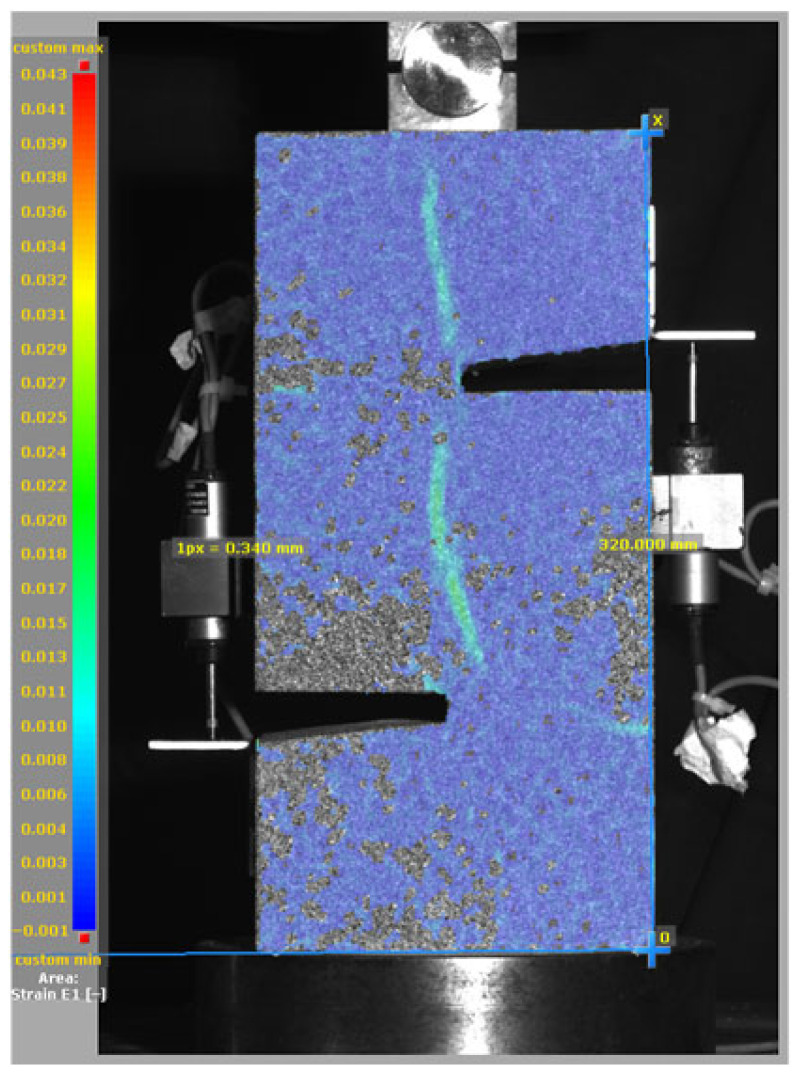	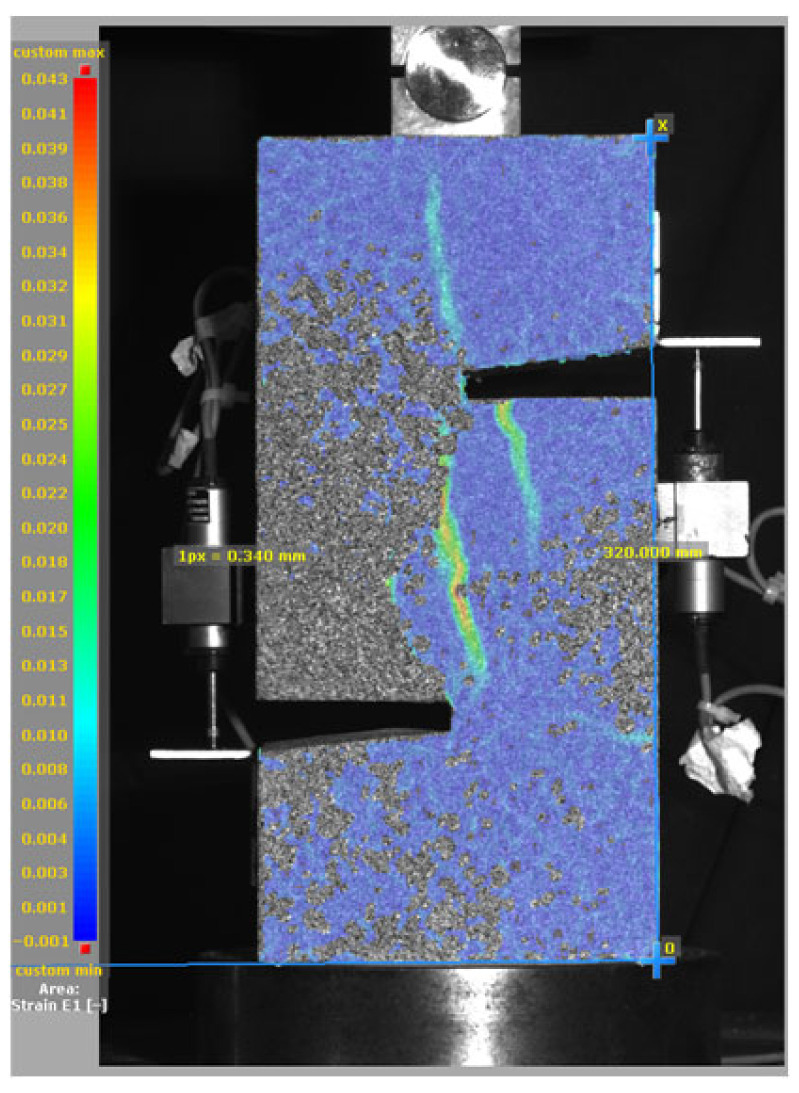
SMA-1.0%-H	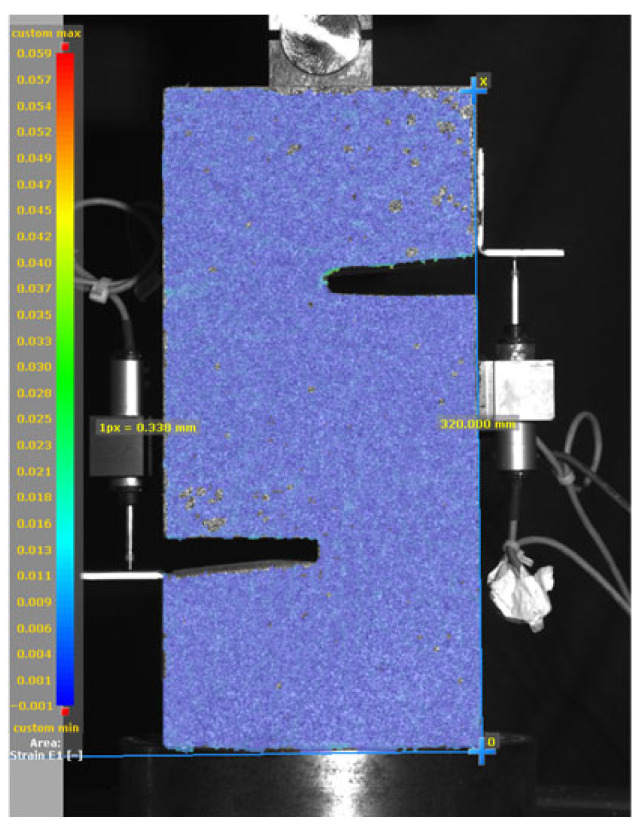	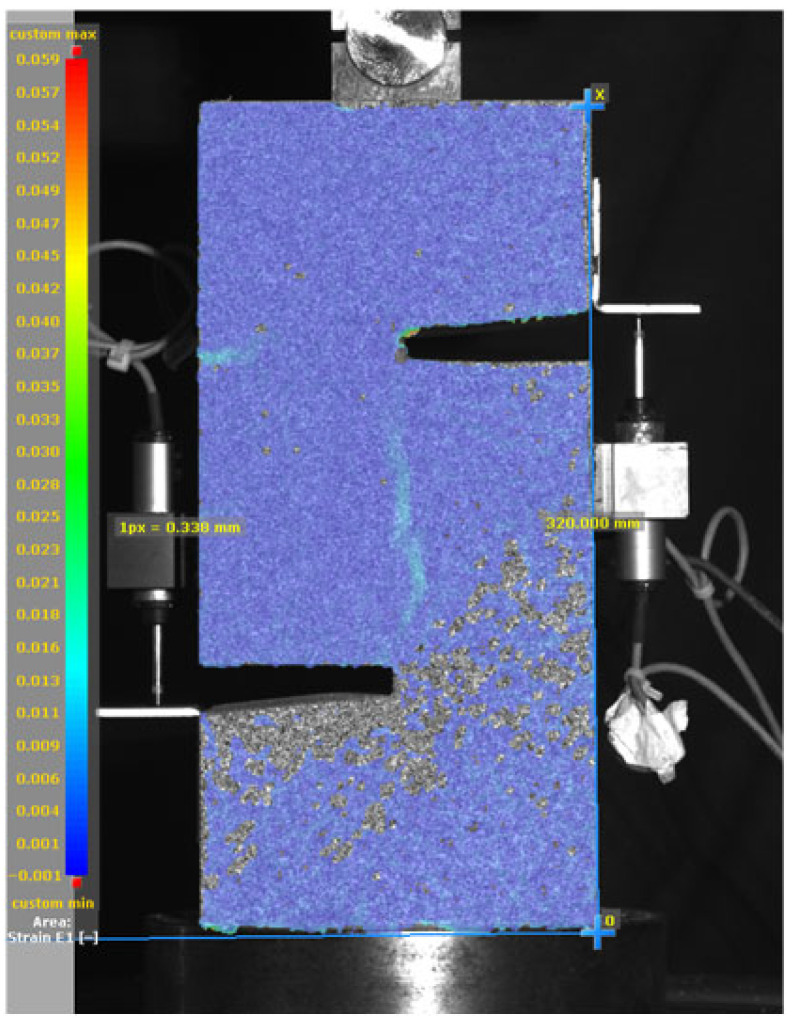	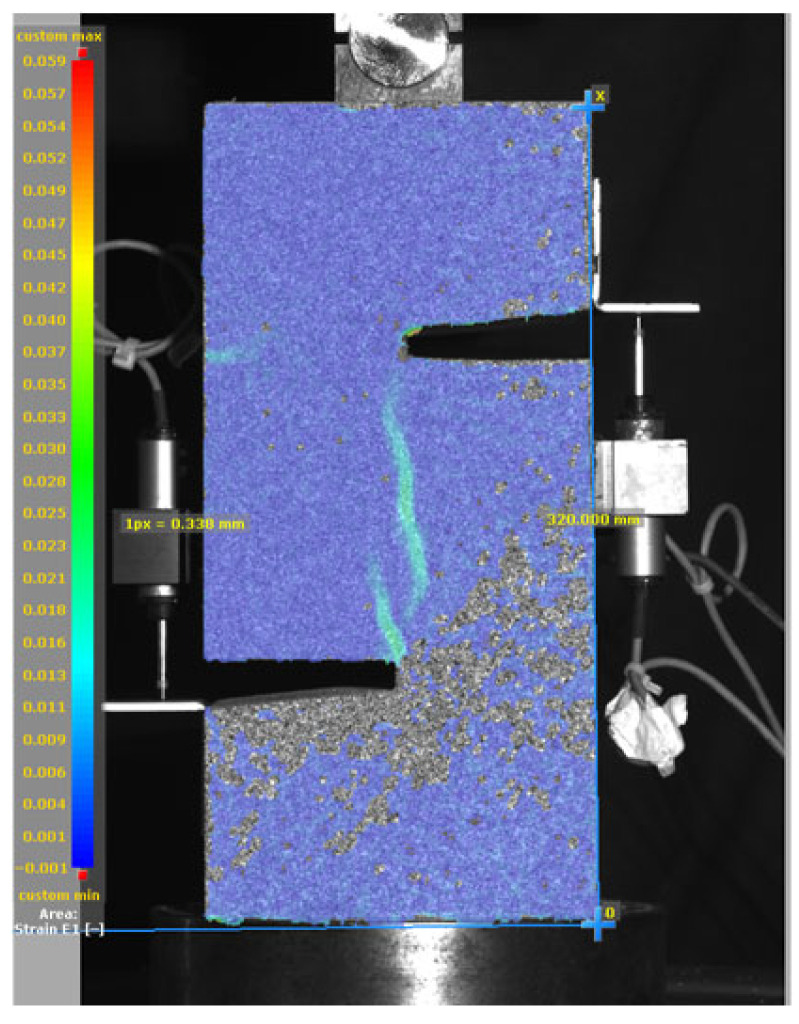	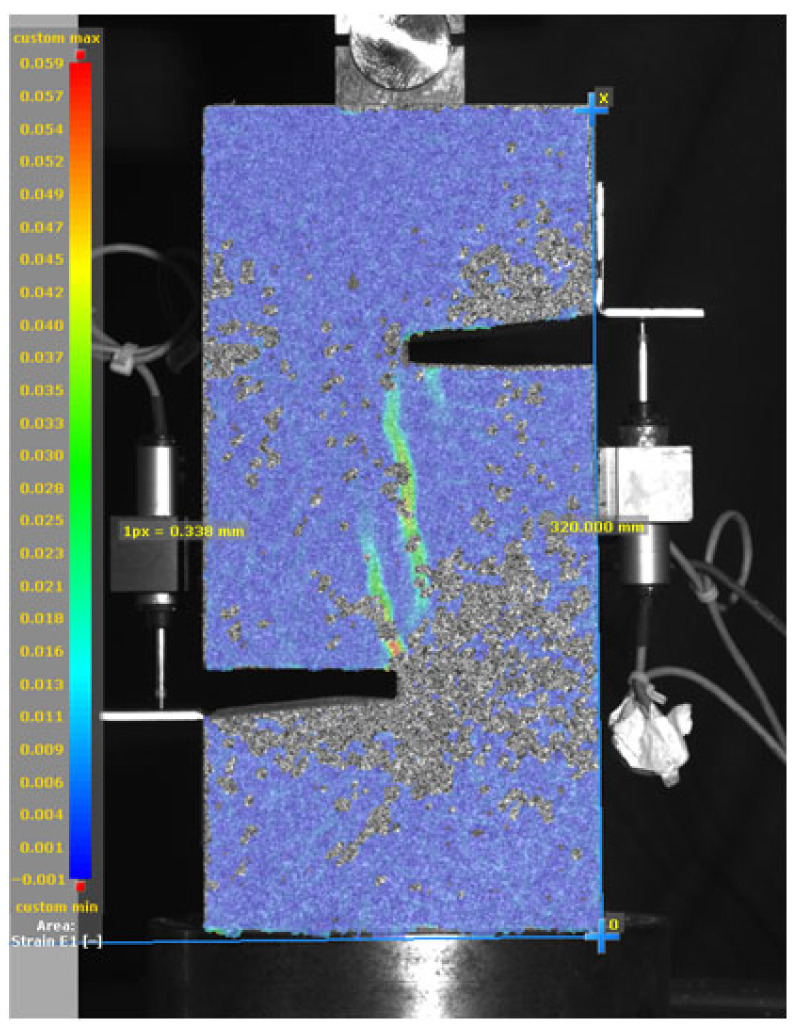
SMA-1.25%-NH	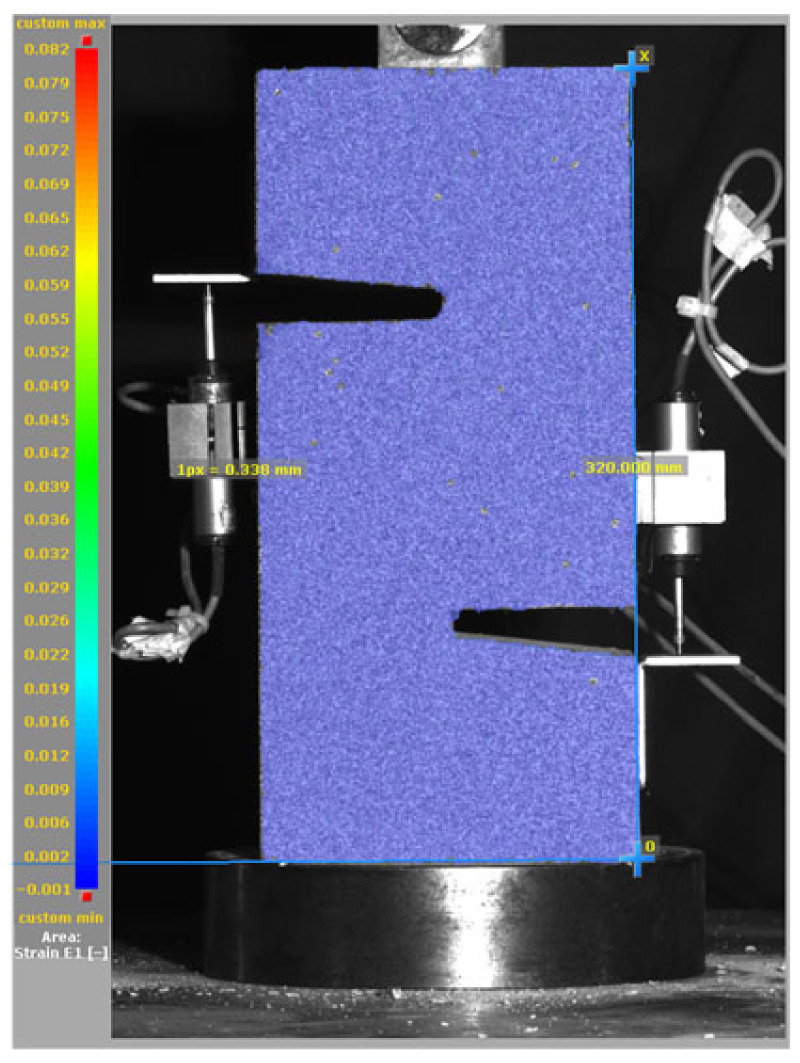	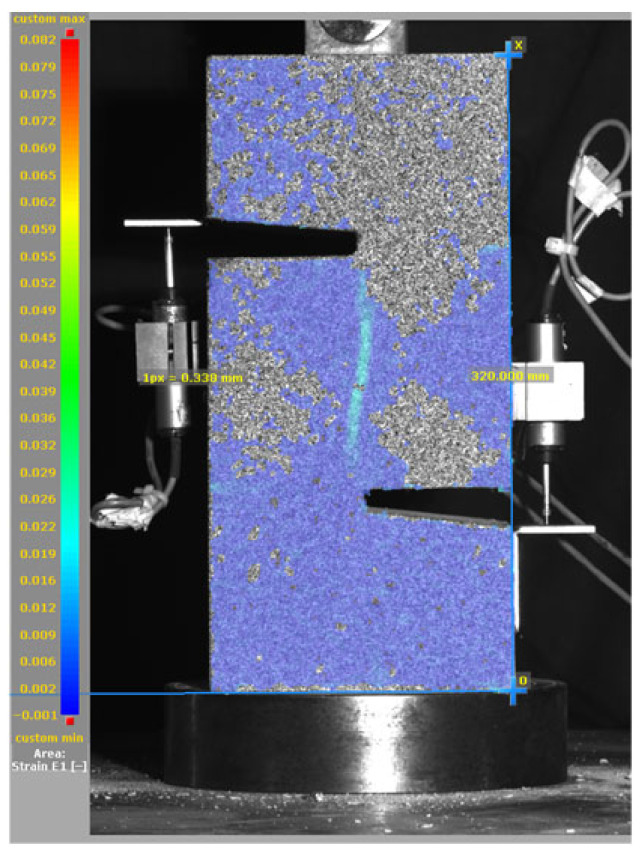	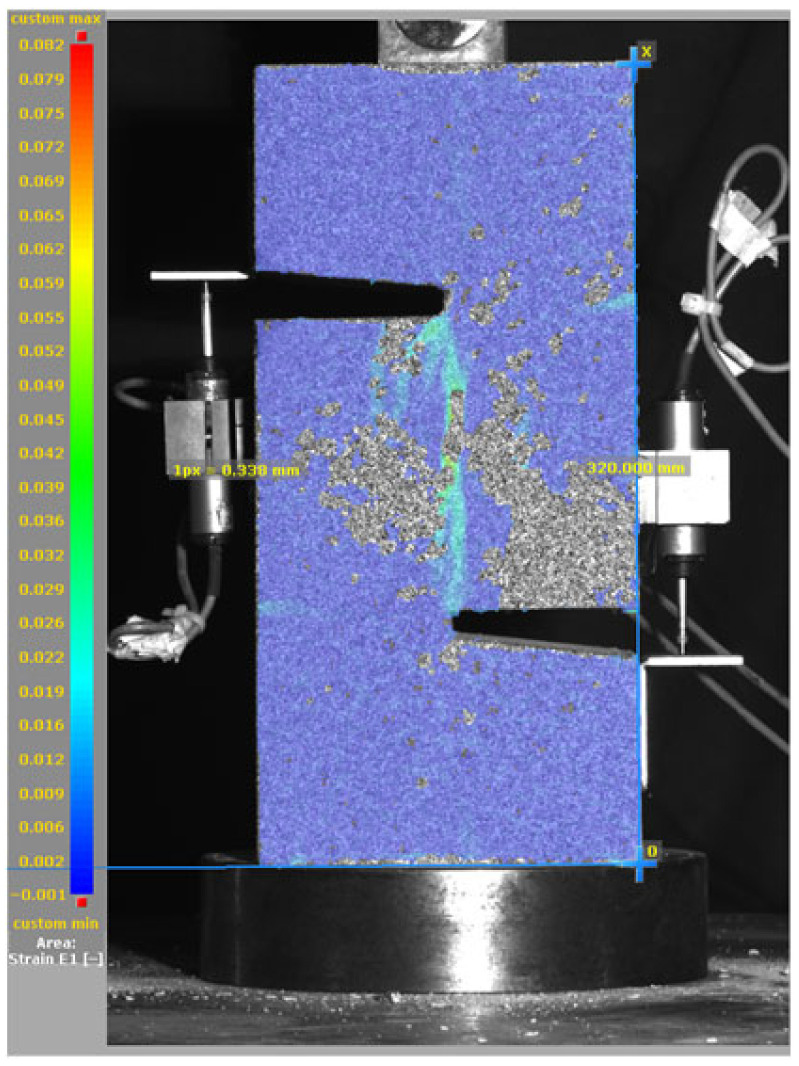	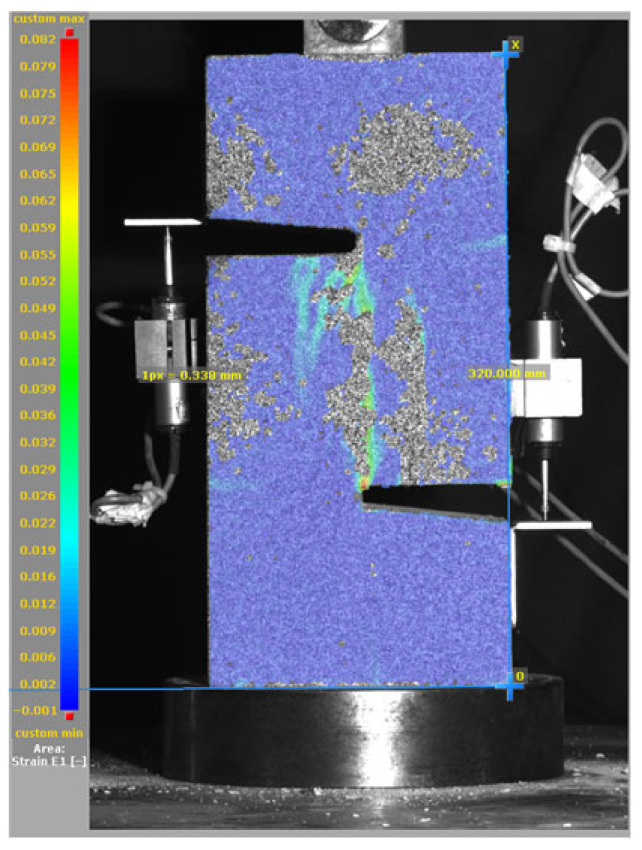
SMA-1.25%-H	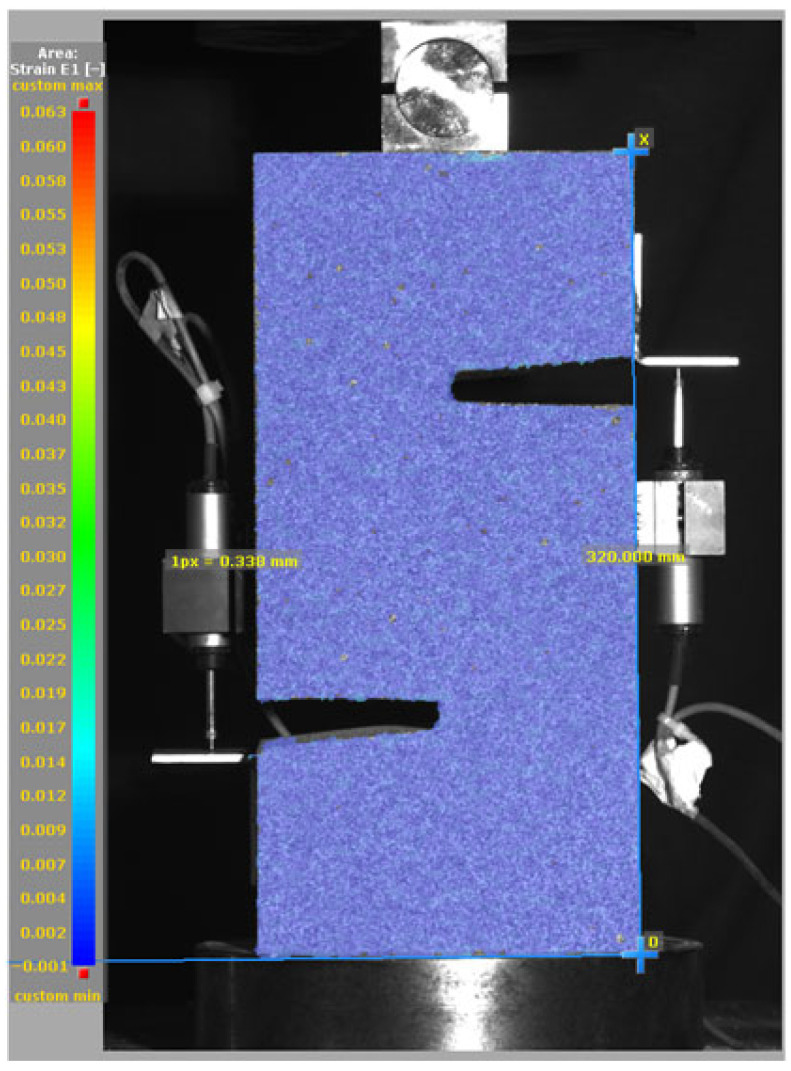	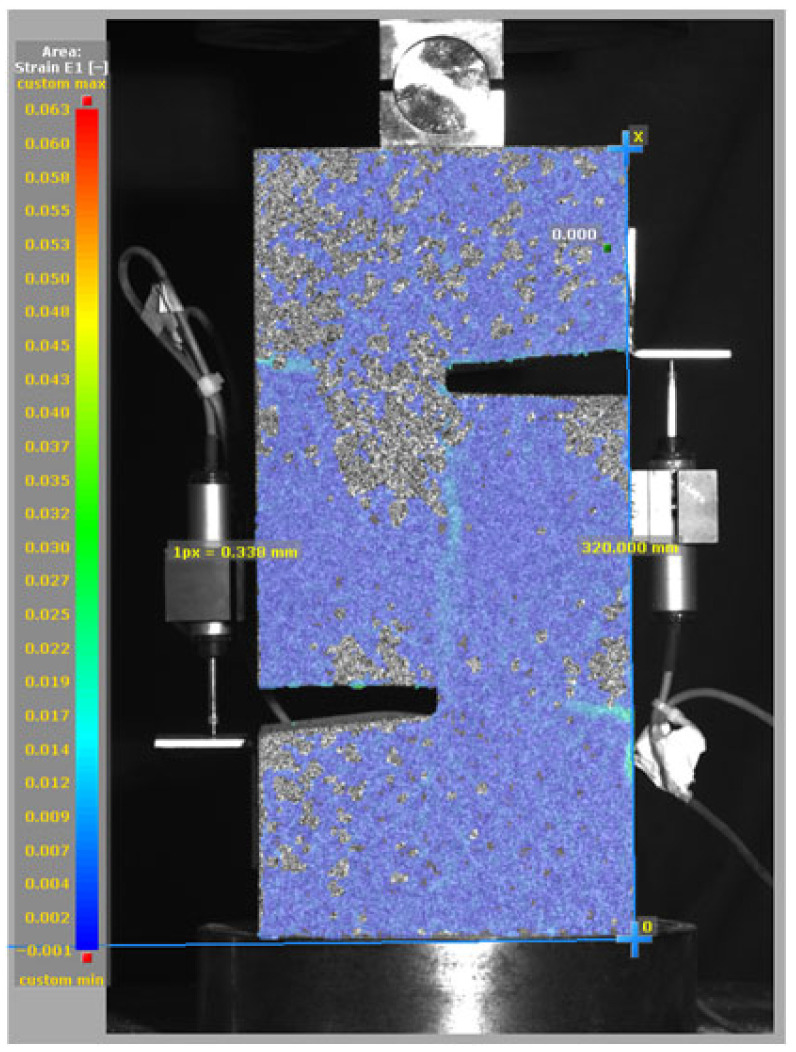	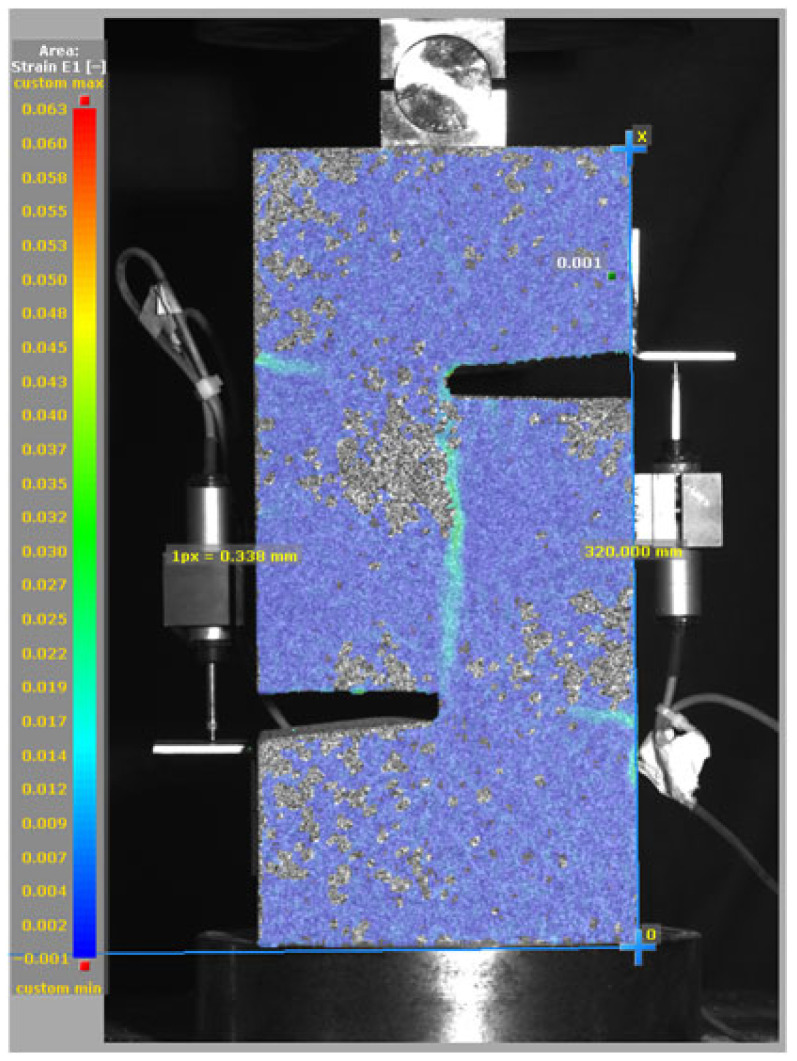	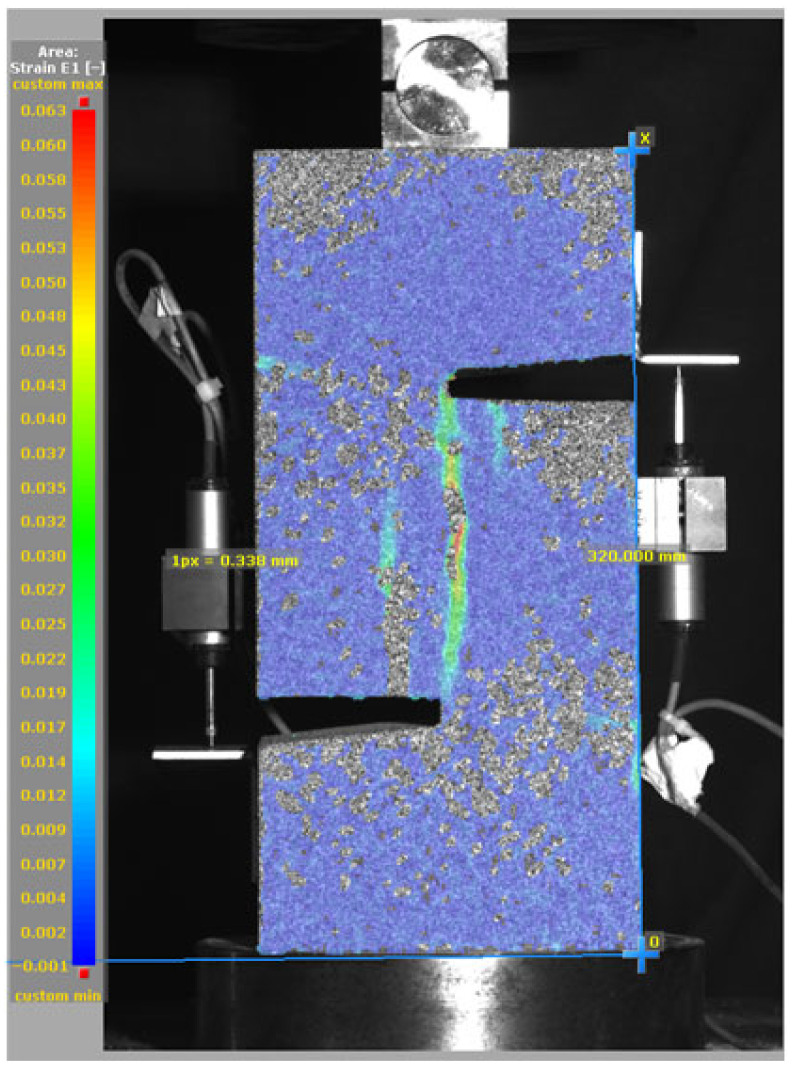

## Data Availability

The data supporting the findings of this study are contained within this article. Further data are available upon reasonable request from the corresponding author.
